# Transcriptomics Indicates Active and Passive Metronidazole Resistance Mechanisms in Three Seminal *Giardia* Lines

**DOI:** 10.3389/fmicb.2017.00398

**Published:** 2017-03-17

**Authors:** Brendan R. E. Ansell, Louise Baker, Samantha J. Emery, Malcolm J. McConville, Staffan G. Svärd, Robin B. Gasser, Aaron R. Jex

**Affiliations:** ^1^Faculty of Veterinary and Agricultural Sciences, The University of MelbourneMelbourne, VIC, Australia; ^2^Population Health and Immunity Division, Walter and Eliza Hall Institute of Medical ResearchMelbourne, VIC, Australia; ^3^Bio21 Molecular Science and Biotechnology Institute, The University of MelbourneMelbourne, VIC, Australia; ^4^Department of Cell and Molecular Biology, Uppsala UniversityUppsala, Sweden

**Keywords:** messenger RNA, RNA sequencing (RNA-Seq), Giardia, metronidazole resistance, oxidoreductases, single nucleotide polymorphism, Trichomonas

## Abstract

*Giardia duodenalis* is an intestinal parasite that causes 200–300 million episodes of diarrhoea annually. Metronidazole (Mtz) is a front-line anti-giardial, but treatment failure is common and clinical resistance has been demonstrated. Mtz is thought to be activated within the parasite by oxidoreductase enzymes, and to kill by causing oxidative damage. In *G. duodenalis*, Mtz resistance involves active and passive mechanisms. Relatively low activity of iron-sulfur binding proteins, namely pyruvate:ferredoxin oxidoreductase (PFOR), ferredoxins, and nitroreductase-1, enable resistant cells to passively avoid Mtz activation. Additionally, low expression of oxygen-detoxification enzymes can allow passive (non-enzymatic) Mtz detoxification via futile redox cycling. In contrast, active resistance mechanisms include complete enzymatic detoxification of the pro-drug by nitroreductase-2 and enhanced repair of oxidized biomolecules via thioredoxin-dependent antioxidant enzymes. Molecular resistance mechanisms may be largely founded on reversible transcriptional changes, as some resistant lines revert to drug sensitivity during drug-free culture *in vitro*, or passage through the life cycle. To comprehensively characterize these changes, we undertook strand-specific RNA sequencing of three laboratory-derived Mtz-resistant lines, 106-2ID_10_, 713-M3, and WB-M3, and compared transcription relative to their susceptible parents. Common up-regulated genes encoded variant-specific surface proteins (VSPs), a high cysteine membrane protein, calcium and zinc channels, a Mad-2 cell cycle regulator and a putative fatty acid α-oxidase. Down-regulated genes included nitroreductase-1, putative chromate and quinone reductases, and numerous genes that act proximal to PFOR. Transcriptional changes in 106-2ID_10_ diverged from those in 713-r and WB-r (*r* ≤ 0.2), which were more similar to each other (*r* = 0.47). In 106-2ID_10_, a nonsense mutation in nitroreductase-1 transcripts could enhance passive resistance whereas increased transcription of nitroreductase-2, and a MATE transmembrane pump system, suggest active drug detoxification and efflux, respectively. By contrast, transcriptional changes in 713-M3 and WB-M3 indicated a higher oxidative stress load, attributed to Mtz- and oxygen-derived radicals, respectively. Quantitative comparisons of orthologous gene transcription between Mtz-resistant *G. duodenalis* and *Trichomonas vaginalis*, a closely related parasite, revealed changes in transcripts encoding peroxidases, heat shock proteins, and FMN-binding oxidoreductases, as prominent correlates of resistance. This work provides deep insight into Mtz-resistant *G. duodenalis*, and illuminates resistance-associated features across parasitic species.

## Introduction

*Giardia duodenalis* (syn. *G*. *lamblia, G*. *intestinalis*) is a parasitic protist of the human gastrointestinal tract that causes 200–300 million clinical cases of diarrheal disease annually (Lane and Lloyd, 2002). The nitroheterocyclic drug metronidazole (Mtz) is routinely used in treatment of microaerophilic parasites including *G. duodenalis, Trichomonas vaginalis* and *Entamoeba histolytica*, and is also used against the anaerobic bacterial pathogens *Helicobacter pylori* and *Clostridium difficile* (Samuelson, 1999; Petri, 2003; Löfmark et al., 2010). Mtz is thought to enter cells as an inactive pro-drug, which is reduced by oxidoreductase enzymes to form cytotoxic intermediates (radicals) that oxidize biomolecules. The low reduction potential of this drug makes it selectively active against microaerophilic and anaerobic organisms, which maintain highly reduced, oxygen-poor intracellular environments. The aerobic host is protected from Mtz cytotoxicity, as any reduced Mtz is spontaneously re-oxidized to the inactive pro-drug form, termed futile cycling (Edwards, 1993). Clinical Mtz resistance has been attributed to increased Mtz tolerance in *G. duodenalis* isolated from patients who failed Mtz therapy (Lemée et al., 2000; Adagu et al., 2001). Similar clinical resistance is reported in *T. vaginalis* (Cudmore et al., 2004) and *H. pylori* (Bereswill et al., 2003; Mirzaei et al., 2014). In addition to studies in clinical isolates, Mtz resistant *G. duodenalis* lines can be derived in the laboratory, which allows genetically controlled (i.e., isogenic) investigation of resistance mechanisms. Such lines are generally created through chronic exposure of drug-susceptible trophozoites to progressively increasing concentrations of Mtz over a period of months (Boreham et al., 1988).

Molecular Mtz resistance mechanisms can be classified as passive or active. Down-regulation of enzymes that reduce Mtz to toxic intermediates, constitute passive resistance mechanisms. Up-regulation of enzymes that detoxify Mtz directly or manage Mtz-induced damage, are considered active mechanisms. Ferredoxin family enzymes, which bind iron-sulfur (Fe-S) clusters, are strongly implicated in both passive and active resistance mechanisms. A well-established passive resistance mechanism centres on pyruvate:ferredoxin oxidoreductase (PFOR) and its electron acceptor ferredoxin. PFOR transfers glycolytic electrons via bound Fe-S clusters to soluble ferredoxin, which can, in turn, activate Mtz (Ellis et al., 1993). Accordingly, lower PFOR and ferredoxin enzyme activities are reported in laboratory-derived resistant lines, relative to their susceptible parents; and PFOR activity correlates with Mtz sensitivity in clinical isolates (Smith et al., 1988; Ellis et al., 1993; Leitsch et al., 2011). Nitroreductase-1, a ferredoxin-nitroreductase chimera, activates Mtz in recombinant enzyme assays, and is transcriptionally down-regulated in resistant lines (Müller et al., 2007b, 2013). Over-expression of nitroreductase-1 in susceptible trophozoites also increases Mtz sensitivity (Nillius et al., 2011). A less direct, passive resistance mechanism involves down-regulation of oxygen detoxification enzymes, which allows intracellular oxygen to accumulate and to inactivate Mtz via futile redox cycling (Ellis et al., 1993). This reaction creates reactive oxygen species, however, which require active detoxification (Testa et al., 2011). In contrast, nitroreductase-2, a paralog of nitroreductase-1, is thought to detoxify Mtz by reducing the Mtz pro-drug directly to an inert amine, thus bypassing the reactive intermediates (Müller et al., 2013, 2015). Transcriptional up-regulation of nitroreductase-2 in particular resistant lines is consistent with an active resistance mechanism. Regulation of thioredoxin reductase (TrxR), a major thiol-cycling enzyme in *G. duodenalis*, is inconsistent in resistant lines. This enzyme provides reducing power to thioredoxins and peroxiredoxins to manage oxidative stress. Over-expression of TrxR in susceptible trophozoites increases Mtz sensitivity, suggesting a role in drug activation (Leitsch et al., 2016); however, increased thiol reductase activity (attributable to TrxR) is reported in some resistant lines, suggesting a protective role (Smith et al., 1988). These conflicting results may be due to the antioxidant activity of TrxR outweighing its detrimental Mtz-activating effects in lines selected for resistance. Reversion to drug sensitivity in formerly resistant lines after passage through the life cycle (i.e., encystation-excystation) or in drug-free culture medium suggests that Mtz resistance is relatively unstable. That nitroreductase-1 and PFOR-1 transcription are restored in revertant excysted trophozoites, further suggests that transcriptional plasticity contributes prominently to Mtz resistance (Müller et al., 2008).

Three well-characterized Mtz-resistant lines are BRIS/83/HEPU/106-2ID_10_ (referred to hereafter as 106-r), BRIS/83/HEPU/713-M3 (713-r), and WB-M3 (WB-r), which were derived in the 1980s through chronic exposure to Mtz. 713-r and WB-r were also exposed to ultraviolet (UV) irradiation prior to selection (Boreham et al., 1988; Townson et al., 1992). Lower PFOR and ferredoxin activities are reported in 106-r, 713-r, and WB-r relative to their susceptible parents (Smith et al., 1988; Townson et al., 1996; Liu et al., 2000; Leitsch et al., 2011), with one study failing to detect any PFOR activity in 106-r (Leitsch et al., 2011). Nitroreductase-1 transcription is down-regulated in 713-r, and nitroreductase-2 is up-regulated both this line and 106-r, indicating that passive and active resistance mechanisms can co-occur within resistant lines (Müller et al., 2013). Chromosomal aberrations are also reported for all three resistant lines, but have not been resolved in detail (Upcroft et al., 1992; Townson et al., 1994; Chen et al., 1995; Upcroft and Upcroft, 1999; Upcroft et al., 2010). Further, 106-r remains infective in suckling mice, whereas 713-r lacks any infective capacity (Tejman-Yarden et al., 2011).

Variation in the infectivity and molecular phenotypes of these resistant *G. duodenalis* lines (reviewed in Ansell et al., 2015b) suggest that multiple molecular resistance phenotypes are possible, each of which could comprise changes in the genetic sequence, transcription level and functional regulation of multiple proteins. Indeed, the extent and variability of molecular phenotypes that confer resistance are far from understood. Some obvious candidates that are yet to be investigated in resistant *G. duodenalis* lines include a canonical nitroreductase NTR-1, that is similar to the RdxA of *H. pylori* and activates Mtz *in vitro* (Nixon et al., 2002; Pal et al., 2009); and several under-characterized NADPH-dependent oxidoreductases that might account for the decreased flavin reductase activity reported in certain lines (Ellis et al., 1993; Leitsch et al., 2011). Further, as we have argued previously (Ansell et al., 2015b), enzymes that produce and consume the substrates and cofactors of oxidoreductase enzymes may thereby modulate their function, which potentially implicates a host of metabolic enzymes in Mtz metabolism and resistance. Recent whole genome and transcriptome sequencing has refined and expanded the complement of genes associated with Mtz resistance in *H. pylori* (Binh et al., 2015) and *T. vaginalis* (Bradic et al., 2016), among which hitherto under-characterized flavin reductases, ribosomal proteins, and canonical nitroreductases feature prominently.

In order to better understand the transcriptional correlates of Mtz resistance in *G. duodenalis* and to probe the features of laboratory-derived resistant lines that may be clinically relevant, the present study used RNA sequencing to compare transcriptional changes in 106-r, 713-r, and WB-r relative to their susceptible parental lines. In addition to identifying differentially transcribed genes (DTGs) and gene sets, we documented single-nucleotide polymorphisms in transcripts that may affect protein function and identified genes that may be negatively regulated by antisense transcription. A core set of resistance-associated genes is discussed in the context of isotype-specific changes that may augment resistance and account for differences in growth rate and infectivity. Central roles for heat shock proteins, peroxidase enzymes and FMN-dependent oxidoreductases in Mtz resistance are underscored via quantitative comparisons of orthologous gene transcription in *T. vaginalis*. This work represents the first genetically controlled, transcriptome-wide investigation of Mtz resistance in any pathogen.

## Materials and methods

### Cell culture

*Giardia duodenalis* trophozoites were generously provided by Professor Jacqueline Upcroft and Professor Peter Upcroft. Metronidazole (Mtz)-sensitive trophozoites (BRIS/83/HEPU/106, BRIS/87/HEPU/713, and WB1B; referred to hereafter as 106-s, 713-s, and WB-s) were maintained in flat-sided 10 mL tubes (Nunclon delta) filled with 10 mL of complete TYI-S33 medium (Davids and Gillin, 2011) and sub-cultured twice weekly. The standard medium contains 55 mM glucose, which is well in excess of the concentration required to sustain trophozoites throughout a four-day culture passage. As such, with a view to correlating transcriptomic results with future metabolomics results (in which glucose can saturate spectral profiles), the TYI-S33 medium was modified to contain 6 mM glucose (designated “TYI medium”). Mtz-resistant lines (BRIS/83/HEPU/106-2ID_10_, BRIS/83/HEPU/713-M3 and WB1B-M3; hereafter 106-r, 713-r, and WB-r) were maintained under the same conditions with Mtz (Sigma Aldrich; 100 mM stock dissolved in dimethyl sulfoxide) added to a final concentration of 30 μM. References describing the original axenization of these lines and resistance selection methods are provided in Table 1.

**Table 1:** **Metronidazole sensitivity and generation times for isogenic lines**.

**Strain**	**Abbreviation**	**References**	**Mtz IC_50_ (μM)**	**IC_50_ fold increase**	**Doubling time (hours)**	**Doubling time fold increase**
106	106-s	Boreham et al., 1984	9.39	–	5.48	–
106-2ID10	106-r	Boreham et al., 1988	23.99	2.55	6.36	1.16
713	713-s	Capon et al., 1989	7.79	–	5.27	–
713-M3	713-r	Townson et al., 1992	42.33	5.43	7.46	1.41
WB1B	WB-s	Capon et al., 1989	8.28	–	5.28	–
WB1B-M3	WB-r	Townson et al., 1992	22.79	2.75	13.8	2.62

### Growth rate and drug sensitivity testing

Trophozoites from confluent flasks were chilled on wet ice (5 min), pelleted (680 × *g*, 5 min, 4°C), diluted in fresh TYI medium to 5 × 10^4^ cells/mL (susceptible lines) or 1.5 × 10^5^ cells/mL (resistant lines; cf Tejman-Yarden et al., 2011), and added in 40 μL volumes to wells of a 96-well clear-bottom plate (Corning #3610). 200 μL of sterile water was added to the peripheral wells to limit evaporation. Serial dilutions of Mtz were created in TYI medium and added to wells containing trophozoites, in duplicate. The wells of the resulting plates contained ~2,000 (susceptible) or 6,000 (resistant) trophozoites in a 50 μL volume, and either Mtz (2.5–200 μM) or 1% DMSO in TYI medium (negative control). Plates were incubated at 37°C within GasPak EZ anaerobe pouches, with fresh anaerobic sachets for 48 h. Reconstituted CellTiter-Glo reagent (Promega) was stored at −20°C and thawed to room temperature (22–24°C; RT) 30 min prior to use; 50 μL of reagent were added to wells containing trophozoites, and plates were incubated for 15 min, shaking, at RT. ATP-based luminescence (corresponding to live cells/well) was measured using a luminometer (BioTek), and transformed to a proportion of the negative control value. Prism software (GraphPad) was used to fit Hill plots to dose-response data and to calculate IC_50_ values, via the “log(inhibitor) vs. normalized response—variable slope” module. In order to estimate generation times, standard curves relating ATP luminescence and trophozoite numbers were created for Mtz-susceptible and -resistant lines, and found to be log-linear within 5 × 10^4^–10^6^ cells per well. Trophozoite numbers in the negative control wells of dose-response experiments were interpolated from the standard curve, and generation times were calculated according to Boreham et al. (1984).

### Sample generation and mRNA sequencing

For Mtz-susceptible lines, samples for mRNA sequencing were generated in triplicate. Falcon t25 flasks were seeded with 10^5^ trophozoites in 56 mL of fresh TYI medium and incubated at 37°C for ~60 h, at which time trophozoites formed a confluent monolayer on the flask walls. For Mtz-resistant lines, up to 8 flasks were seeded with 1.5–3 × 10^6^ trophozoites (according to growth-rate), and Mtz was added to a final concentration of 30 μM. After incubation, flasks were inverted, and supernatant and suspended trophozoites (likely to contain dead/dying and dividing cells, which could confound the mRNA integrity and comparative transcription analyses, respectively), were decanted to waste; 50 mL of ice-cold complete TYI medium were added and flasks were incubated on ice-water for 10 min to detach trophozoites. The suspended cells were transferred to 50 mL falcon tubes and pelleted (680 × *g*, 5 mins, 4°C). Supernatant was removed, and pellets were re-suspended, transferred to UV-irradiated 1.5 mL microfuge tubes, and pelleted (720 × *g*, 2 mins, RT). Samples from Mtz-resistant lines were consolidated into three microfuge tubes. Supernatant was removed and pellets were dissolved in 1 mL of TriPure reagent (Roche), incubated at RT for 5 min and stored at −80°C.

RNA was extracted according to the TriPure manufacturer's protocol within 4 weeks of sample preparation. The dried RNA pellet was re-suspended in reverse-osmosis deionized water (H_2_O) and treated with Turbo DNAse (Ambion) according to the manufacturer's instructions. RNA concentration was estimated by fluorometry (Qubit), and quality control was performed using a BioAnalyzer (Agilent). Polyadenylated RNA was purified from 10 μg of total RNA using Sera-mag oligo(dT) beads, fragmented to a length of 100–500 bases, and reverse transcribed using random hexamers. Strands were labeled using the dUTP second-strand synthesis method, end-repaired and adaptor-ligated, and then treated with uracil-specific excision reagent (USER; NEB) before amplification by PCR. Products were purified over a MinElute column (Qiagen) and single-end sequencing was performed using an Illumina HiSeq 2500 (YourGene Biosciences, Taiwan). Raw RNA sequencing reads are available through the NCBI Sequence Read Archive (http://www.ncbi.nlm.nih.gov/sra/SRP075868).

### Data processing and analysis

Raw reads were trimmed using Trimmomatic (Lohse et al., 2012) (sliding window: 4 nt; minimum average PHRED quality: 20; leading and trailing: 3 nt; minimum read length: 40 nt), and mapped as single-ended reads to the accepted *G. duodenalis* gene models (assemblage A genome release 25; giardiaDB.org; Morrison et al., 2007) using RSEM (Li and Dewey, 2011) with the -forward-prob 0 flag. The locus encoding superoxide reductase (Testa et al., 2011) was added to the reference genome. Reads that did not map under these conditions were likely to include antisense transcripts. To quantify antisense transcription, unmapped reads were submitted to RSEM with the –forward-prob 1 flag, which discards any reads that map with <100% confidence to the coding strand. The RSeQC bam_stats.py module (Wang et al., 2012) was used to calculate read mapping statistics; and infer_experiment.py was used to confirm read mapping orientation. Feature detection was calculated as a function of read mapping depth using the Qualimap counts module (v1.0; García-Alcalde et al., 2012) with the −k 10 flag, denoting a minimum mapped read threshold of 10. Saturation plots were displayed in Excel (Microsoft) (Supplementary Figure 1).

Expected counts from RSEM were submitted to edgeR (Robinson et al., 2009). For preliminary clustering analyses, low abundance genes (fewer than one count per million counts; CPM) were discarded before library re-scaling and TMM normalization. For each susceptible and resistant pair, sample normalization was performed as above and differentially transcribed genes were determined at a false discovery rate of 0.01. CPM-values were multiplied by 1,000 and divided by the reference transcript length to give transcripts per kilobase per million transcripts (TPM), suitable for comparing the transcriptional abundance of different genes across samples. Romer (Wu et al., 2010) was used to test enrichment of KEGG pathways and Gene Ontology (GO) terms among up- and down-regulated genes in resistant lines, and to test enrichment of curated gene sets specific to *G. duodenalis* (significance threshold: corrected *p* < 0.05; Supplementary Table 9). Hypothetical *G. duodenalis* proteins of interest were selected for tertiary structure prediction using a stand-alone implementation of the I-TASSER software suite (v3.0) generously provided by Professor Yang Zhang, as described previously (Ansell et al., 2016). It must be noted that the output from the stand-alone implementation of this software can differ from that available through the online server (zhanglab.ccmb.med.umich.edu/I-TASSER) due to computational resource constraints on the server. As such, the output from the stand-alone software should be more robust. GO terms associated with high-confidence structures (TM score > 0.5; GO confidence score > 0.5) were included in gene set testing. Structural similarity searching was performed using TM-align (Zhang and Skolnick, 2005), and protein structures were visualized using UCSF Chimera (Pettersen et al., 2004). Read coverage data were normalized by total library size (calculated in edgeR), and transcriptional abundance plots and heat maps were generated in R (ggplot2 and gplots packages).

RNA reads mapping in the sense orientation were submitted to mpileup and bcftools (SAMtools suite; Li et al., 2009) to identify single nucleotide polymorphisms (SNPs) using conservative thresholds (≥100 × read coverage for the alternative allele, constituting ≥20% of nucleotides mapped at that position). Selected SNPs were visually confirmed using Integrative Genomics Viewer (Robinson et al., 2011), and amino acid substitutions (products of non-synonymous SNPs), were predicted using getORF (Rice et al., 2000) and custom scripts. For comparative transcriptomics between species, published RNA transcript count data for Mtz-susceptible and –resistant *T. vaginalis* (Bradic et al., 2016) were submitted to edgeR in a two-sample comparison of transcript abundance. Count data for resistant and susceptible *G. duodenalis* lines generated in this study were compared using the same design, and orthologous genes (defined in OrthoMCL-DB; Chen, 2006) that were differentially transcribed in the same direction in both species, were retained for further analysis.

## Results

### Growth rates and metronidazole susceptibility

**Figure 1:**
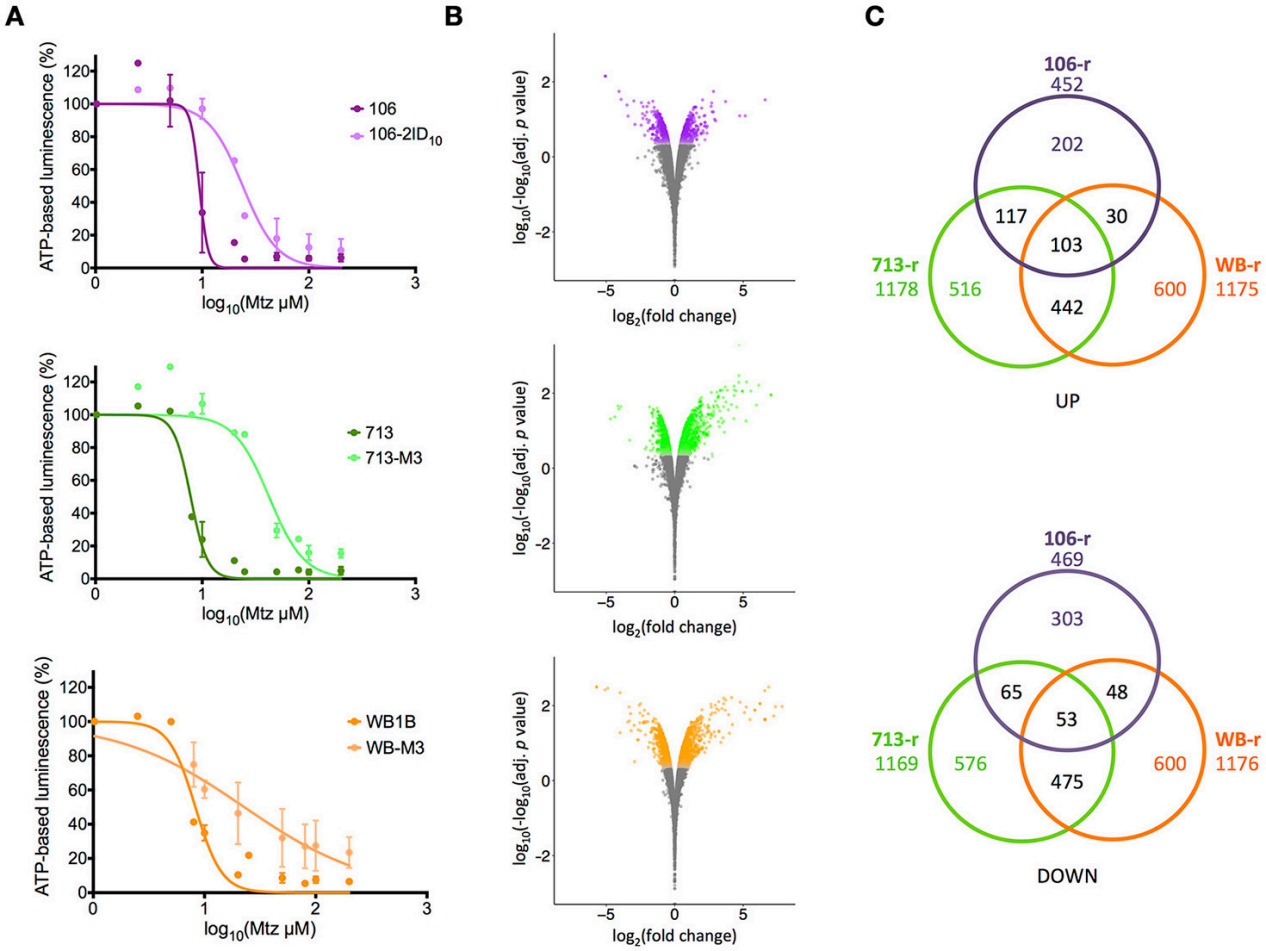
**Metronidazole dose-response and differential transcription in resistant lines. (A)** Dose-response curves for metronidazole-susceptible lines (dark colors) and resistant progeny (light colors). Experiments performed in biological duplicate. Error bars represent ± 1 standard deviation. **(B)** Fold-change in transcriptional abundance in resistant lines relative to susceptible parents. Significantly differentially transcribed genes (corrected *p* < 0.01) are displayed in color. Y-axes represent log-log-transformed corrected *p* values, where higher numbers indicate greater significance. **(C)** Numbers of significantly up-regulated (top panel) and down-regulated (bottom panel) genes shared between resistant lines. The total number of differentially transcribed genes is displayed beneath the line name.

All Mtz-susceptible lines exhibited generation times of ~5 h. The generation time for WB-r was 13.85 h, indicating a marked growth defect compared to 106-r and 713-r, for which generation times were 6.36 and 7.46 h, respectively. For susceptible lines, Mtz IC_50_ concentrations ranged from 7.8 to 9.4 μM. Each resistant line showed significantly greater Mtz tolerance than its susceptible parent (two-sample comparison of fits; *p* < 0.001; Figure 1A). Specifically, WB-r and 106-r exhibited an approximate 2.6-fold increase in Mtz tolerance relative to their susceptible parents, whereas 713-r was 5.4-fold more tolerant than 713-s, and also more tolerant than the other resistant lines (713-r vs. WB-r: *p* = 0.027; vs. 106-r: *p* = 0.004; Table 1).

### Clustering and differential transcription

RNA sequencing of six cell lines in biological triplicate produced a total of 641 million single-ended reads, with an average of 35.6 million reads per sample. The vast majority of reads satisfied quality control (mean = 93.5%; range = 92.4–94.7%), of which 80% were mapped to the accepted *G. duodenalis* genes in the sense orientation (Supplementary Tables 1, 2; Supplementary Figure 1). Around 5% of reads from each sample mapped to the reverse complement, consistent with antisense transcripts (detailed below). After filtering lowly transcribed genes, 5,792 non-deprecated coding domains were detected in all isotypes. Transcripts from a further 231 coding domains were detected in either one or two isotypes (Supplementary Table 3). Using transcript abundance, susceptible lines clustered together in both principal coordinate analysis and Euclidean clustering; although Pearson correlations were lower on average between susceptible lines compared with resistant lines (Pearson's *r* = 0.93 vs. 0.96). Omission of transcripts encoding VSPs improved the mean pair-wise correlation for susceptible lines (*r* = 0.98), with little effect on resistant lines (*r* = 0.97; Supplementary Figure 2).

Pearson correlations based on log_2_(fold-change) values in resistant lines, were strongest between 713-r and WB-r (*r* = 0.47). These lines also correlated with microarray-based fold-change data for 41 strongly differentially regulated genes in the previously published Mtz-resistant line WB-C4 (0.27 < *r* > 0.37; Müller et al., 2008). Transcriptional changes in 106-r diverged substantially from 713-r and WB-r (*r* ≤ 0.2), and correlated negatively with WB-C4 (*r* = −0.29). Comparisons with transcriptomic data for WB-s trophozoites after exposure to sub-lethal stressors (Ansell et al., 2016) revealed strongest agreement between Mtz stress and 106-r (*r* = 0.18), and a modest correlation with 713-r (*r* = 0.03). At a false-discovery threshold of 0.01, 2,010 genes (i.e., non-deprecated coding domains) were up-regulated in at least one resistant line relative to the susceptible parent, and 2,120 genes were down-regulated (Figure 1B; Supplementary Tables 4, 5). In 713-r, 1,178 genes were up-regulated, and 1,169 were down-regulated relative to 713-s. In WB-r, 1,175, and 1,176 genes were up- and down-regulated respectively, of which 917 were differentially transcribed in the same direction in 713-r. Comparatively few DTGs (921 in total) were identified for 106-r, supported by close clustering between the resistant and susceptible lines for this isotype (Supplementary Figure 2B; overlapping DTGs are displayed in Figure 1C). This result may also be affected by greater variance between replicates in 106 (mean biological co-efficient of variance = 0.18) compared to WB and 713 isotypes (mean BCV = 0.1). As such, the minimum change in transcript abundance for genes satisfying the statistical threshold for differential transcription in 106-r was 24%, compared to 17% in WB-r and 713-r. The 30 most strongly up- and down-regulated genes in each resistant line (excluding VSPs) are provided in Supplementary Table 6.

### Common differentially transcribed genes and mutations

**Figure 2:**
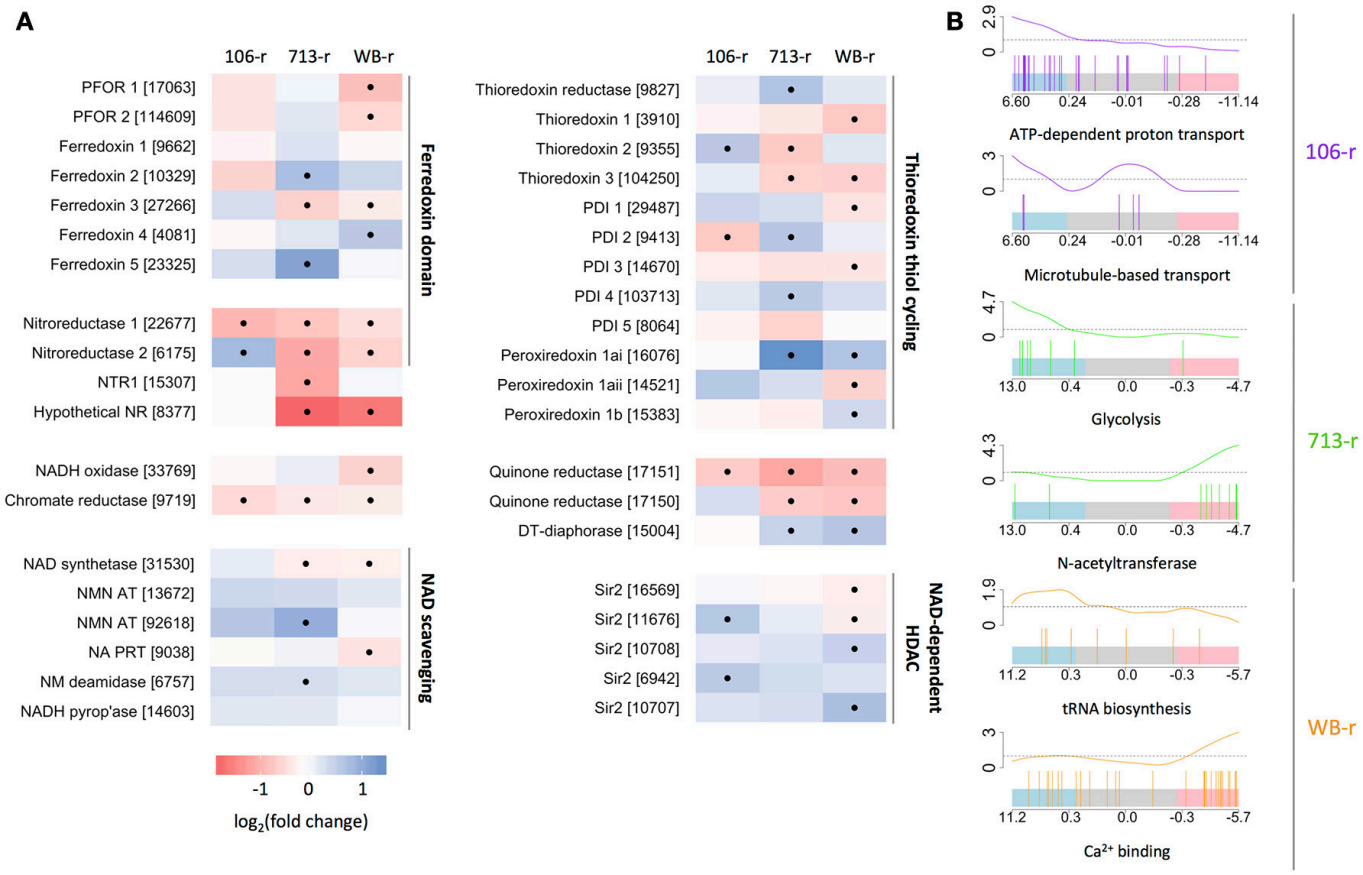
**Transcriptional changes in oxidoreductase-coding genes and related genes. (A)** Color-scaled log_2_(fold change) values for resistant lines. Black dots indicate a statistically significant difference. Genes are grouped based on structure and/or function. Accession numbers (prefix: GL50803) appear in brackets. **(B)** Bar-code plots indicating the rank and log_2_(fold change) of genes (high: left, to low: right) comprising selected gene sets, below curves displaying sliding enrichment ratios. Blue and red shading indicate upper and lower quartiles, respectively.

**Figure 3:**
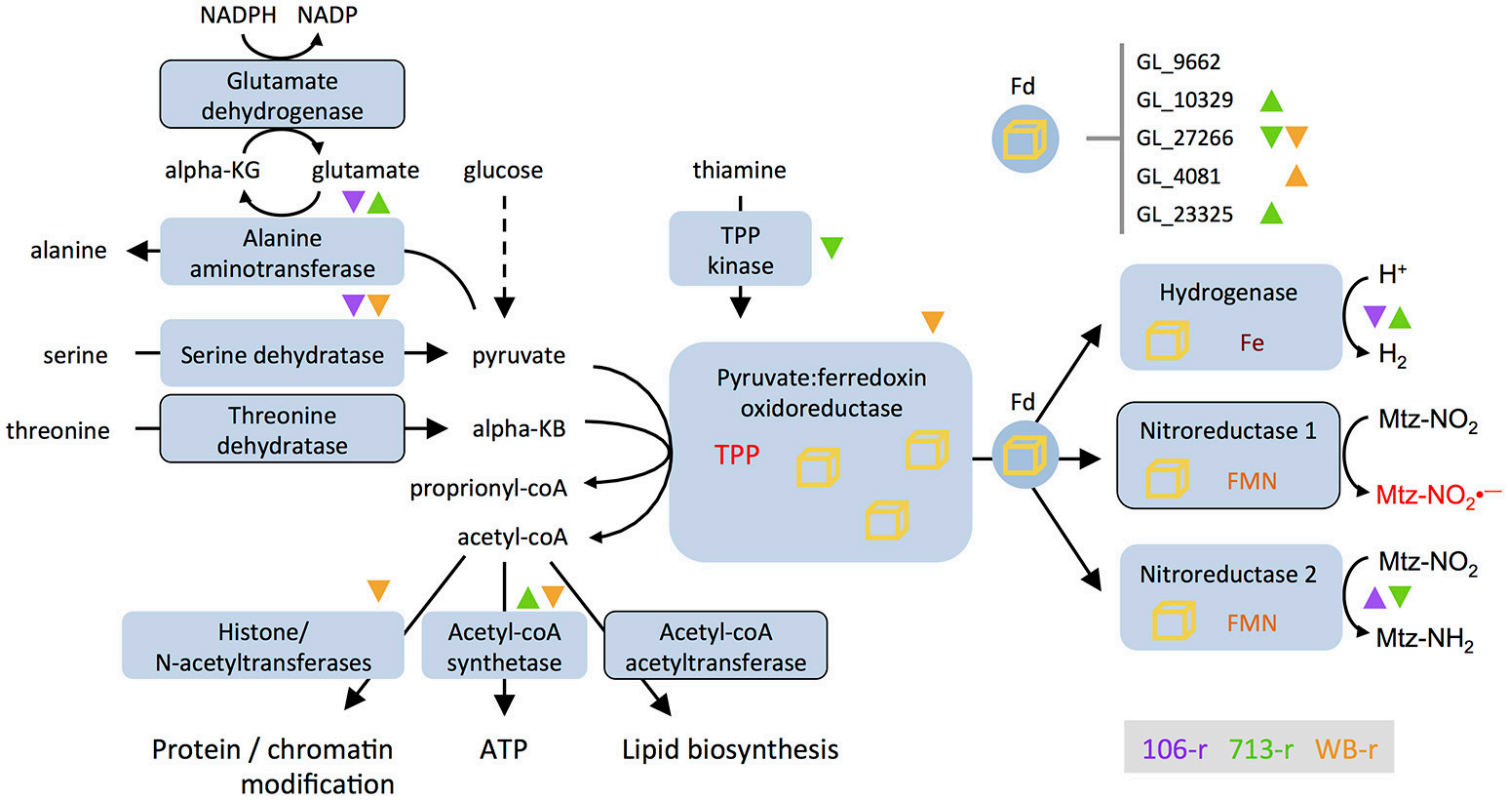
**Differential transcription of genes involved in ferredoxin-based electron transport and related processes**. Nitroreductases are depicted as substrates of ferredoxin (Fd), as postulated by Ali and Nozaki (2007). The glutamate shunt consumes NADPH and cycles glutamate and α-ketoglutarate to drive the conversion of pyruvate to alanine. Enzymes encoded by genes that are down-regulated in all three resistant lines are outlined. Direction of differential transcription in different lines is indicated with color-coded up- or down-pointing triangles. Differential transcription for five ferredoxin-coding genes appears at top-right. Iron sulfur clusters are depicted as yellow cubes; Nitroreductase-1 activates metronidazole to toxic intermediates (Mtz-${\text{NO}}_{2}^{•-}$, red). Nitroreductase-2 reduces Mtz to an inert amine (Mtz-NH_2_). KG, ketoglutarate; KB, ketobutyrate; coA, co-enzyme A; TPP, thiamine pyrophosphate; FMN, flavin mononucleotide; Fe, iron.

Of 156 DTGs common to all three resistant lines, 103 genes were up-regulated, and 53 were down-regulated (Figure 1C). Common up-regulated genes included 33 VSPs, a high-cysteine membrane protein, the mitotic regulator Mad2 (Vicente and Cande, 2014), zinc and calcium transporters, Nek and DYRK kinases, a sulfatase-like enzyme, and a putative fatty acid α-oxidase. A ParcC-like chaperone (GL_15455), and two putative mTor kinase subunits were also up-regulated (GL_6542 and GL_13469). The 53 common down-regulated genes included nitroreductase-1, putative FMN-dependent chromate and quinone oxidoreductases (GL_9719 and GL_17151), a putative calcium sensor, glutamate dehydrogenase, threonine dehydratase, acetyl co-A acetyltransferase, two α-giardins, and E1 and E2 ubiquitin-related enzymes (Figure 2A; Figure 3; Supplementary Table 5). Proton- and ATP-dependent transporters, a hexose transporter and a putative sugar-nucleotide transporter (GL_9036) were also down-regulated. In all resistant lines, threonine dehydratase and a small hypothetical protein (GL_104062) encoded different non-synonymous SNPs. A further 19 genes encoded identical substitutions in two of three resistant lines (Table 2; Supplementary Table 7; Supplementary Figure 3).

**Table 2:** **Predicted effects of non-synonymous SNPs in metronidazole-resistant lines**.

**Substitution**	**Accession no**.	**Description**	**106-r (aa)**	**713-r (aa)**	**WB-r (aa)**	**PDB chain**	**TM score**	**RMSD (Å)**	**Species**
	GL_2834	ARF GAP		**I291V**	**I291V**				
	GL_99228	Cytochrome P450 2E1	T70A		T10I	3e6iA	0.5953	3.46	*Homo sapiens*
	GL_8682	Glucose-6-phosphate 1-dehydrogenase	G638E	T339A					
	GL_101589	High cysteine protein		N168H, N215D, R224Q	Q268K, Q269P, T270K				
	GL_102180	High cysteine protein	**I173V**		**I173V**, I175T				
	GL_103944	Nek kinase		N165S, V171I, I190M	N165S				
	GL_42657	Nek kinase		**R578H**	**R578H**				
	GL_16839	Nek kinase		**N289D**	**N289D**				
	GL_20593	Neuronal acetylcholine receptor subunit α-4		**M58V**	**M58V**	2llyA	0.7027	2.98	*Homo sapiens*
	GL_98178	Neuronal acetylcholine receptor subunit α-7	Q61H		R82H	2mawA	0.744	2.49	*Homo sapiens*
	GL_114625	Proprotein convertase precursor	**S37P, V41A, K188E, N193S, C195R**		**S37P, V41A, K188E, N193S, C195R**				
	GL_8496	Rac/Rho-like protein		**K79N**	**K79N**				
	GL_31921	Tetracycline repressor protein class D		T46M, R55S, **P57S**	**P57S**, S73G, P83Q	2o7oA	0.8263	2.37	*Escherichia coli*
	GL_12108	Threonine dehydratase (LTC)	N12D	D348H	L739P				
	GL_9121	TON 1535		**N54K**	**N54K**	3zpjA	0.8206	2.06	*Thermococcus onnurineus*
	GL_104062	Tyrosyl-tRNA synthetase	D94G	A104V, L108P	F39L	2cycA	0.6109	3.86	*Pyrococcus horikoshii*
	GL_20592	Ubiquitin conjugation factor E4	**D55N**, D56G		**D55N**	2qj0A	0.5448	4.59	*Saccharomyces cerevisiae*
	GL_101496	VSP		**E53K**, S70G, S71N, **V342A**	**E53K, V342A**				
	GL_101765	VSP		E275A, **T435A**, I544T, T560A	**T435A**				
	GL_113093	VSP	A49D, N51K		P62T, S63P				
	GL_113491	VSP	A144T	D60G, **P60T**, **A64S**	**P60T, A64S**				
	GL_137710	VSP	**T410K**, G417E	**E249D, T410K**	**E249D**				
	GL_40571	VSP		**A211S, M216I, I216V, N223T**	**A211S, M216I, I216V, N223T**				
	GL_101074	VSP		N679S	N679S				
	GL_92835	VSP		V43I, V44A	G47D, N48S, A49S, S135T, S136C				
	GL_8623	Cleavage stimulation factor subunit		C91T		2ooeA	0.8828	2.3	*Mus musculus*
	GL_22677	Nitroreductase-1	C583T						
	GL_5947	Ribosomal protein L35a	A1C						
	GL_9173	Putative S/T protein kinase pkwA		C349T					
	GL_18725	Transcriptional regulator, TetR family	**C269A**	**C269A**		3eupA	0.7651	2.77	*Cytophaga hutchinsonii*
	GL_31366	Type III effector XopAI		C377A		4elnA	0.6173	3.34	*Xanthomonas citri*
	GL_42357	VP1		G1237T		3cnfB	0.8476	2.52	*Bombyx mori cypovirus 1*
	GL_111732	VSP		C111A					

*Identical amino acid substitutions and nonsense mutations, are printed in bold. LTC, lateral transfer candidate. aa, amino acid; nt, nucleotide; Putative structures were predicted for hypothetical proteins. Annotations and alignment metrics are derived from the closest empirically determined structural homologues. TM score indicates overall agreement between structures (0–1); RMSD, root mean squared deviation (Å) in central carbon position between structures*.

### *Giardia*-specific membrane proteins in resistant lines

According to previous studies, resistant lines may preferentially express certain VSPs (Chen et al., 1995; Müller et al., 2007a, 2008). As mentioned above, disparities in VSP transcription preferentially decreased the strength of correlation between susceptible lines. When the 20 most abundant VSP-coding transcripts (approximating the top decile) in each line were compared, there was greater convergence in resistant lines. Ten genes among the top decile in at least one resistant line (including GL_137617, which was in the top decile for all three resistant lines), were absent from the equivalent group in all susceptible lines. A further nine genes were among the top decile in all resistant lines, whereas only six genes were consistently highly transcribed in susceptible lines (Supplementary Figure 4). No VSPs were consistently suppressed in resistant- relative to susceptible lines. The VSP TSA417 (GL_113797), which was reported as transcriptionally suppressed in the Mtz-resistant lines WB-C4 and WB-C5 (Müller et al., 2007a, 2008), was among the top decile of VSPs in 713-s, and in both 106-s and 106-r. VSPAS7 (GL_137740), which was suppressed in WB-C4 (Müller et al., 2008), was in the top decile for WB-r only. CRP136 (GL_103142), was previously detected in WB-r but not WB-s (Chen et al., 1995). In this study however it was among the top VSP decile in both lines. Overall, these results show little agreement in VSP transcription between lines, and within lines over time. To test the possibility that particular VSP peptide sequences are under selection in the presence of Mtz, we investigated the proportion of clade I, II, and III VSPs among the top decile in resistant and susceptible lines (Adam et al., 2010). Clade II was over-represented in both resistant and susceptible lines, relative to the background prevalence, with little between-group difference (odds ratio = 1.44 and 1.65, respectively). Similarly, there was little difference in the number of CXXC motifs (average of 18–21 motifs), or cysteine content (9–10% cysteine) in the top decile of *vsp*s between lines (Supplementary Table 8). High cysteine membrane proteins are another prominent *Giardia*-specific protein family with variable numbers of cysteine motifs (Davids et al., 2006). Transcripts from 60 *hcmp*s were significantly enriched among up-regulated genes in 713-r, but not 106-r, or WB-r. A clade of six *hcmp*s whose transcription tended to increase in all resistant lines contained the only consistently up-regulated HCMP, GL_11309. Sixty-nine CXXC motifs are encoded in this gene, well above the median for the HCMP family (Davids et al., 2006). No HCMPs were consistently down-regulated (Supplementary Figure 5).

### Gene set enrichment and isotype-specific DTGs in Mtz-resistant *Giardia*

#### 106s vs. 106-r

**Figure 4:**
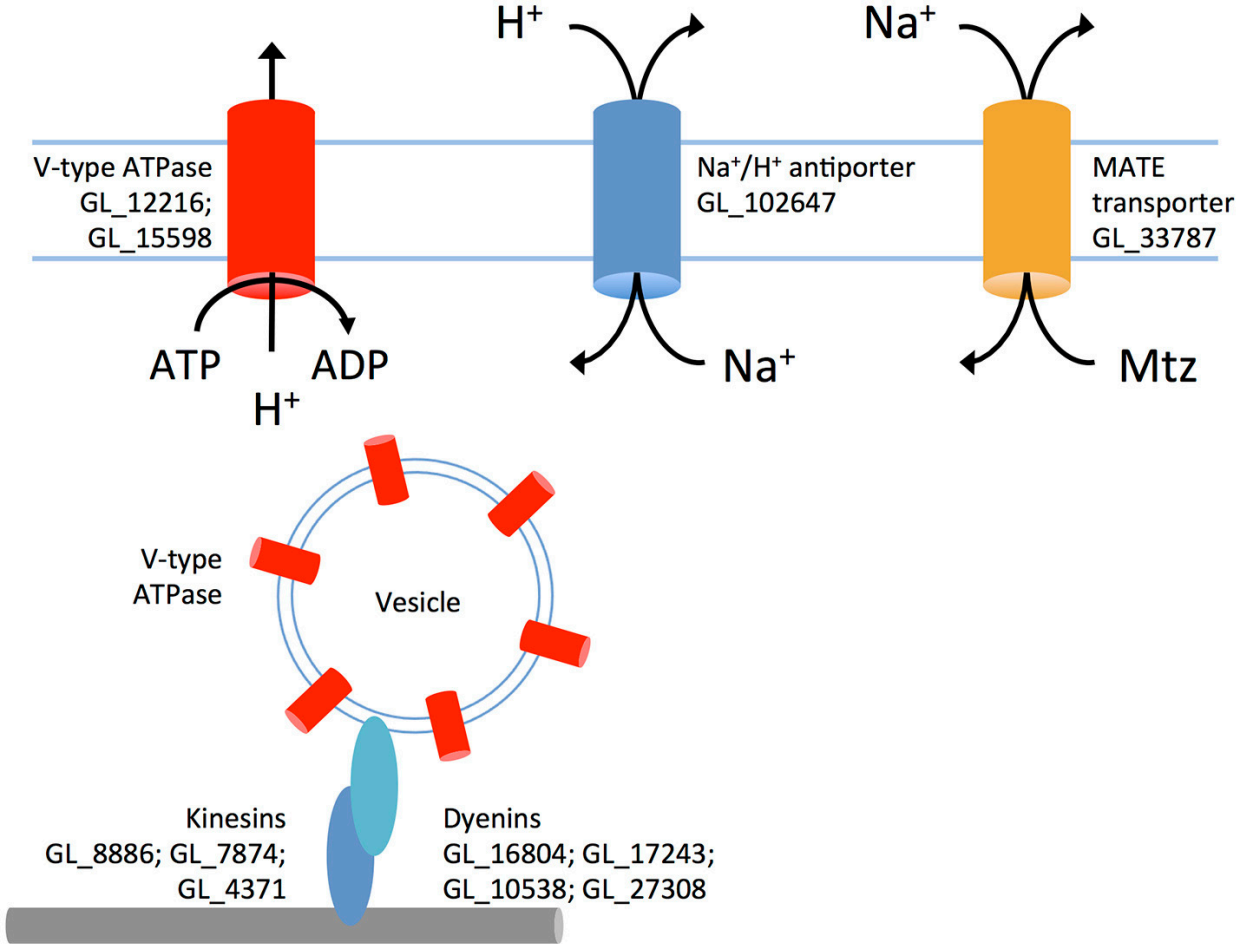
**A putative metronidazole efflux system in 106-r**. V-type ATPases are inserted into vesicular membranes and transported via microtubules and dynein/kinesin motors to the plasma membrane. These pumps use ATP to transport protons out of the cytosol against the diffusion gradient. An up-regulated sodium/proton antiporter exchanges in-flowing protons for sodium, increasing the extracellular sodium concentration. The MATE transporter is proposed to expel metronidazole as sodium flows back into the cell. Up-regulated genes (prefix GL50803 abbreviated to GL) are displayed next to proteins.

In agreement with previous reports, nitroreductase-2 was up-regulated in 106-r (Müller et al., 2015). Whereas, qPCR analysis had indicated non-significant down-regulation of nitroreductase-1-coding transcripts in 106-r (Müller et al., 2013), this effect was statistically significant in our data. The previously reported ablation of PFOR activity in 106-r (Leitsch et al., 2011) did not correspond to down-regulation of PFOR-coding transcripts. Gene set enrichment testing revealed increased transcription of the KEGG NAD synthesis pathway, and five NAD-dependent Sir2 enzymes in 106-r. Genes annotated with GO “ATP hydrolysis coupled proton transport,” namely vacuolar ATPase components, were also up-regulated, as was GO “microtubule-based movement,” contributed by dyenin- and kinesin-coding transcripts (Figure 2B; Figure 4). Conversely, gene sets encoding tRNA synthesis enzymes, protein chaperones, and FAD-binding enzymes were suppressed. The latter result derived from lower transcription of glutamate synthase, NADH oxidase (Brown et al., 1996) and dihydrouridine synthase. The KEGG arginine biosynthesis pathway was also down-regulated, contributed by lower transcription of genes in the ATP-forming arginine dihydrolase pathway (Schofield et al., 1992). A complete list of differentially transcribed gene sets is provided in Supplementary Tables 9–12. Among the 202 DTGs uniquely up-regulated in 106-r were a MATE-like sodium antiporter, Gmyb11 and the cell-cycle related proteins cyclin-fold protein, cell division control protein-48, and a CDK regulatory subunit. Among 303 uniquely down-regulated genes were the transcription factor Myb1, a putative Tet repressor homolog (GL_32769), iron hydrogenase, 15 high-cysteine membrane proteins, and α-giardins 8, 12, and 14. Intriguingly, a premature stop codon was identified in 29% of transcripts mapped to *nitroreductase-1* (nucleotide substitution C583T; Supplementary Figure 6).

#### 713-s vs. 713-r

Nitroreductase-1 transcription was down-regulated in 713-r, in accordance with previous work. However nitroreductase-2 was not transcriptionally up-regulated as indicated previously (Müller et al., 2013). Gene sets encoding protein chaperones (HSPs, TCP-1 subunits, and peptidyl-prolyl isomerases), ABC and cation transporters, and the glycolysis pathway, were enriched among up-regulated genes in 713-r. Up-regulation of a set of genes encoding 4Fe-4S cluster-biding proteins, was founded on increased transcription of ferredoxin-2, two un-annotated ferredoxins (GL_23325 and GL_4081), and a putative glutamate synthase (GL_87577). Ferredoxin-3, which is predicted to bind iron-sulfur clusters of a 2Fe:2S geometry, was significantly down-regulated in 713-r—a finding that may relate to previous reports of suppressed ferredoxin activity (Liu et al., 2000; Leitsch et al., 2011). Gene set testing also identified down-regulated N-acetyltransferases and iron-binding proteins, the latter contributed by three putative ferretins (Figure 2B). A curated set of FMN-binding genes was found to be down-regulated, which included the putative quinone reductase (GL_17150), NTR-1 and the putative canonical nitroreductase GL_8377 (Figure 2A). Conversely, peroxiredoxin-1ai and flavohaemoglobin were strongly induced in 713-r. Predicted amino acid substitutions unique to 713-r were identified in thymidine kinase, GL_17150 (D37A) and its paralog DT-diaphorase (Y69H), CMGC and calcium/calmodulin-like kinases, serine dehydratase, and a putative transcription factor (GL_31921) among other proteins (summarized in Supplementary Table 7).

#### WB-s vs. WB-r

Despite relatively close agreement in the transcriptional changes observed in 713-r and WB-r detailed above, a number of unique differentially transcribed gene sets were identified in WB-r. tRNA biosynthesis genes and the KEGG N-glycan biosynthesis pathway were up-regulated, whereas a set of genes encoding calcium-binding enzymes were down-regulated, contributed by lower transcription of three calmodulins, calcineurin, five group E annexins (α-giardins), and annexins of group 6 and A2. Further, iron-binding proteins were down-regulated, incorporating four putative lipoxygenases, an Fe-S cluster scaffold, and both PFOR paralogs. The latter finding correlates with the decline in PFOR activity previously reported in WB-r (Townson et al., 1996). In addition, WB-r was the only resistant line in which the oxygen detoxification enzyme NADH oxidase (Brown et al., 1996), was significantly down-regulated. Given the markedly slower growth of WB-r compared to 106-r and 713-r, we investigated the transcripts that were differentially transcribed in 106-r and 713-r, to the exclusion of WB. Up-regulated genes in this group included a sulfatase maturating enzyme, a putative uracil DNA glycolsylase implicated in base excision repair, a ClpB chaperone, and dynein- and kinesin-related proteins. Also up-regulated were fructose bisphosphate aldolase, one of two glyceraldehyde 3-phosphate dehydrogenases (GL_6687), and the previously mentioned putative glutamate synthase (GL_87577). Genes down-regulated in 106-r and 713-r to the exclusion of WB-r included glucosamine 6-phosphate N-acetyltransferase, a MAF1-like RNA-polymerase III repressor, ubiquitin ligase E3A, and a proteasomal regulatory subunit. Further, a highly-transcribed putative tRNA methyltransferase homologue (GL_17245) was only detected in the 106 and 713 isotypes, whereas a number of short hypothetical proteins were exclusively detected in WB lines (Supplementary Table 3). Finally, non-synonymous mutations in transcripts encoding glucose-6-phosphate dehydrogenase (Table 2), and a truncation in GL_18725, were unique to 106-r and 713-r. No truncations were predicted in the WB-r proteome.

### Characterization of putative NAD(P)H oxidases

**Figure 5:**
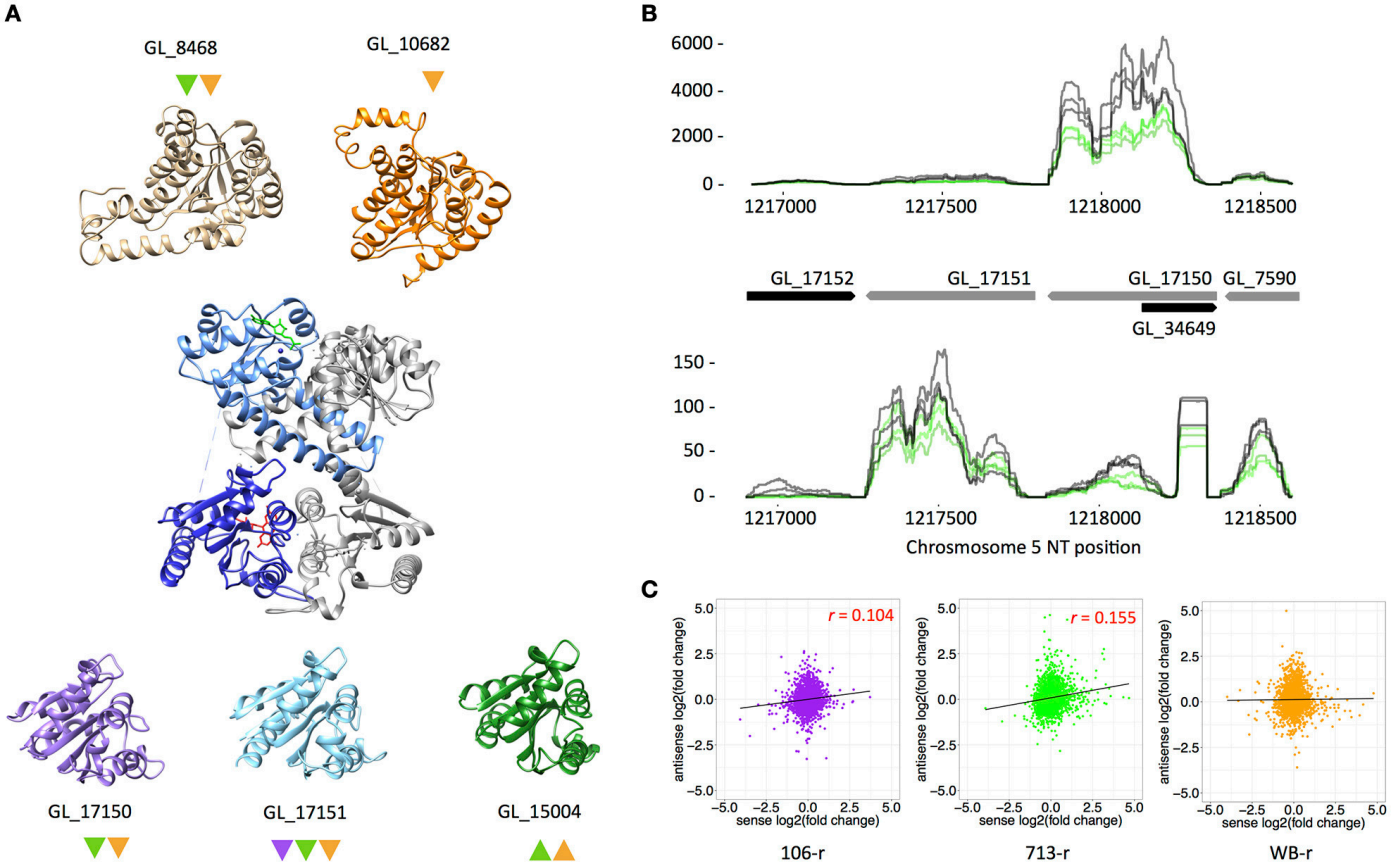
**Putative structures for FMN-dependent quinone reductases and transcriptional regulation. (A)** Transcripts encoding a quinone-reductase like enzyme (GL_17151) are down-regulated in all resistant lines. Predicted structures for this enzyme and two paralogs are shown at bottom. The three predicted protein stuctures are similar to the crystal structure of KefF (PDB code 3EYW), displayed at centre. Monomers within dimers forming the Kef complex are coloured dark blue (KefF), and light blue (KefC). Two proteins with a KefC-like fold were identified (at top), of which GL_8468 shows a transcriptional profile similar to GL_17150. Differential transcription of genes encoding these proteins is indicated with color-coded up- or down-pointing triangles (purple, 106-r; green, 713-r; orange, WB-r). **(B)** Sense (top panel) and antisense (bottom panel) transcript abundance over a region in chromosome 5 encoding the paralogous putative quinone reductases, GL_17151 and GL_17150. Normalized read depth for three replicates of 713-s (gray lines) and 713-r (green lines) is displayed, and is representative of other lines. Gene models are displayed in black (forward strand) and gray (reverse strand). **(C)** Pearson correlations of log_2_(fold change) values for sense (x-axis) and antisense (y-axis) transcripts in metronidazole-resistant lines, relative to susceptible parent lines. Positive correlations for 106-r and 713-r are displayed in red.

We undertook further structural and transcriptional investigation of differentially transcribed FMN-dependent NAD(P)H oxidoreductases in resistant lines. GL_9719 and GL_17151 were down-regulated in all resistant lines. The predicted structure of GL_9719 is highly similar to the crystal structures of FMN-binding chromate reductases from *Thermus scotoductus* (Opperman et al., 2010) and *Bacillis subtilis* (Kitzing et al., 2005) (Supplementary Figure 7). The putative quinone reductase GL_17151 has two paralogs in assemblage A *G. duodenalis*: DT-diaphorase, which is up-regulated in 713-r and WB-r; and GL_17150, which is down-regulated in the same lines. The predicted structures of all three proteins are homologous with the KefF (K^+^ efflux regulator) subunit of the *Escherichia coli* KefF/KefC complex (Figure 5A; Roosild et al., 2009). In *E. coli*, KefF associates with a NAD-binding KefC subunit via residues that are not conserved in the predicted *G. duodenalis* homologs. Nevertheless, we identified two hypothetical proteins (GL_8468 and GL_10682) with predicted NADP-binding pockets and structural similarity to KefC. Further, GL_8468 was down-regulated in 713-r and WB-r, in similarity to GL_17150 (Figure 5A). Interestingly, GL_17150 and GL_17151 are adjacent on chromosome 5, suggesting a gene duplication. Whereas these neighbours share only 43% amino acid sequence identity, GL_17150 is 86% identical to DT-diaphorase, which is encoded at a distant locus. Further, GL_17150 is vastly more highly transcribed than either GL_17151 or DT-diaphorase (~800 vs. 40 TPM). To probe a possible mechanism of transcriptional regulation for these paralogous genes, we quantified antisense transcription from GL_17150 and GL_17151. Transcripts antisense to GL_17151 were 4-fold more abundant than those antisense to GL_17150, however the presence of a potentially confounding pseudogene antisense to GL_17150 (GL_34649), should caution the interpretation of these results (Figure 5B).

### Antisense transcription

We further analysed antisense transcription from 2,268 non-overlapping genes that were detected in all isotypes. A total of 792 antisense transcripts were up-regulated across resistant lines, of which 53 were identified in all lines. Alternatively, 703 down-regulated antisense transcripts were identified across resistant lines (34 in all lines; Supplementary Tables 13, 14). A single antisense transcript encoding a solute transporter (GL_9036) was up-regulated in all lines and corresponded with down-regulation of the coding transcript in the same manner. When the fold change in sense and antisense transcripts from non-overlapping genes in resistant lines was compared, positive correlations were identified for 106-r (*r* = 0.104) and 713-r (*r* = 0.155; Figure 5C). This finding corresponds to higher proportions of directionally correlated sense and antisense DTGs in all lines (Supplementary Table 13).

### Pan-specific transcriptional correlates of Mtz-resistance

Two-sample comparisons between Mtz-resistant and -susceptible *G. duodenalis* and *T. vaginalis* revealed orthologous heat shock protein-coding genes, peroxidases and dullard-like phosphatases that were exclusively up-regulated in resistant lines in both species (Supplementary Table 15; Supplementary Figure 8). Two large orthologous groups comprised genes encoding NEK kinase/ankyrin repeat proteins, and protein transport-related molecules, that were up- and down-regulated in both species. Down-regulated orthologous groups contained threonine dehydratases, 40S and 60S ribosomal proteins, the putative *G. duodenalis* chromate reductase GL_9719 and a *T. vaginalis* putative etylmaleimide reductase (TVAG_411220), UTP glucose-phosphate metabolizing enzymes; and the previously mentioned putative quinone reductase (GL_17151) and its ortholog TVAG_311580.

## Discussion

Drug resistance can prevent the effective treatment of parasitic diseases in humans and animals. For neglected infectious diseases such as giardiasis, the problem of drug resistance is compounded by a limited arsenal of anti-parasitic drugs, and limited data concerning both the epidemiological extent, and biochemical mechanisms, of drug resistance. The present study used RNA sequencing to identify DTGs and gene sets, and SNPs in transcripts in Mtz-resistant *G. duodenalis* lines relative to their susceptible parents. We further increased the value of these analyses by incorporating putative structural and functional information for hypothetical proteins using the I-TASSER software suite. Of course it must be noted that predicted functions are just that, and that the true nature of the many hypothetical proteins in *Giardia* awaits experimental determination. Only two reports to date have investigated transcriptional correlates of Mtz resistance using genome-wide techniques (Müller et al., 2008; Bradic et al., 2016). In this study, in addition to sensitive genetically-controlled analyses of transcriptional changes between Mtz-resistant isotypes of *G. duodenalis*, we compared our results to these independently generated data sets to infer key transcriptional themes in Mtz resistant parasites more broadly. Transcriptional changes in lines 713-r and WB-r were relatively similar, whereas changes in 106-r were qualitatively different and comparatively modest. At the gross cellular level, however, the line WB-r grew more slowly than lines 106-r and 713-r, suggesting a higher cost of resistance. These data indicate that divergent molecular phenotypes can produce similar cellular resistance features, and *vice versa*. We attempted to reconcile the transcriptional and cellular phenotypes of the three resistant lines by analyzing both common transcriptional changes, and those unique to each line. The excellent sequencing depth attained in this study also afforded sensitive investigation of SNPs in transcripts, which can overcome the genetic idiosyncrasies of *G. duodenalis*. Namely, trophozoites contain two transcriptionally active diploid nuclei (Kabnick and Peattie, 1990), and in resistant lines, stable aneuploid states are possible (Chen et al., 1995). However, because the sum of actively transcribed alleles is manifest in the transcriptome, inferring the functional effects of SNPs at this level, should better reflect the proteome of resistant cells.

### Functional inhibition of pyruvate:ferredoxin oxidoreductases

The earliest reports of Mtz-resistant *G. duodenalis* identified changed intracellular redox conditions, and perturbed enzyme activities (Gillin and Reiner, 1982; Smith et al., 1988; Ellis et al., 1993). Recurring themes have since emerged, in particular, the association of ferredoxin family proteins with resistance. PFORs operate at the interface of glycolytic, amino acid, and fatty acid metabolism in *G. duodenalis*, and supply electrons to ferredoxins that activate Mtz. Both PFOR paralogs require a thiamine pyrophosphate (TPP) catalytic co-factor, and can decarboxylate pyruvate and oxaloacetate. PFOR-2 can also catabolize α-ketobutyrate (Townson et al., 1996). Transcriptional suppression of genes that act proximal to PFORs in all lines, may impose functionally analogous limits on PFOR activity, and thus Mtz activation. Firstly, PFOR activity may be impaired by lower transcription of serine and threonine dehydratases, which produce pyruvate and α-ketobutyrate, respectively. Transcriptional suppression of thiamine pyrophosphokinase, identified in 713-r, may similarly inhibit PFOR activity by restricting supply of the TPP cofactor. That threonine dehydratase acquired non-synonymous mutations in all lines, and was consistently suppressed, may reflect the lower energetic value of its product (proprionyl-coA), which is a less preferred substrate of the ATP-forming acetyl-coA synthetase (Sanchez and Müller, 1996). Other transcriptional changes suggest that accumulation of pyruvate, which has antioxidant effects in *G. duodenalis* (Biagini et al., 2001b) may be prioritized in resistant lines. Indeed, the suppression of acetyl-coA acetyltransferase in all resistant lines, lower transcription of histone- and N-terminal acetyltransferases in 713-r, and induction of histone deacetylases in 106-r, are all consistent with acetyl-coA conservation (albeit at the cost of lipid metabolism, protein quality control, and epigenetic regulation). This in turn may promote pyruvate accumulation by inhibiting carbon flux through PFOR. In this context, lower glutamate dehydrogenase transcription in all resistant lines might further bolster the pyruvate pool by limiting its consumption through the “glutamate shunt” (Park et al., 1998; Müller, 2003). Glutamate dehydrogenase suppression would also conserve NADPH, a key source of reducing power for antioxidant enzymes such as TrxR (Figure 3).

### FMN-binding oxidoreductases

Down-regulation of the ferredoxin-nitroreductase chimera, nitroreductase-1, was observed in all three lines investigated here, and is reported in the majority of Mtz-resistant *G. duodenalis* investigated to date (reviewed in Ansell et al., 2015b). Recombinant nitroreductase-1 reduces Mtz when NADH is supplied as an electron donor (Müller et al., 2015). This NADH-dependent mechanism is reminiscent of canonical nitroreductases that reduce Mtz at the NADH-binding site (Koder and Miller, 1998; Martinez-Julvez et al., 2012), and may therefore be catalytically independent of the ferredoxin domain. We contend that the toxicity of nitroreductase-1 in Mtz-exposed *G. duodenalis* could be due to an additional mechanism of drug activation that involves electron transport through the ferredoxin domain, via the FMN cofactor, to the NADH-binding site. Indeed, although *G. duodenalis* is widely claimed to lack a ferredoxin:NADH reductase (FNR), such a function has been theoretically attributed to nitroreductase-1 (Ali and Nozaki, 2007; Figure 3). FNR activity in nitroreductase-1 would represent a parsimonious link between pyruvate catabolism and NAD reduction under drug-free conditions (Ellis et al., 1993). By extension, in resistant lines, functional inhibition of PFOR would directly limit nitroreductase activity. The ability of ferredoxin-nitroreductases to accept electrons from PFOR or reduced ferredoxins (as is likely for Fe-hydrogenase; Mulder et al., 2011) and, thereby, to reduce NAD or Mtz, remains to be tested.

Two other putative FMN-dependent oxidoreductases were down-regulated in all resistant lines, indicating potential roles in Mtz activation. The predicted structure of the putative chromate reductase (GL_9719) is similar to that of enzymes with low substrate specificity that reduce xenobiotic electrophilic compounds such as trinitrotolulene (Blehert et al., 1999). The physiological substrates of these enzymes remain elusive, although a chromate reductase in the human gut commensal *Bacilis subtilis*, is induced after exposure to hydrogen peroxide, and proposed to reduce the unsaturated products of peroxidized lipids (oxylipins; Kitzing et al., 2005). In this context, the up-regulation of a putative fatty-acid α-oxidase in all resistant lines is particularly interesting. In plants, tissue damage or chemical insults induce oxidation of the haem co-factor bound in fatty acid α-oxidase (Zhu et al., 2013), which drives the conversion of fatty acids into oxylipins. These modified lipids are potent signaling molecules (Blée, 2002) that drive transcriptional changes (Eckardt, 2008) and are implicated in regulating growth, stage conversion and pathogenicity in numerous parasitic protists (reviewed in Noverr et al., 2003). Taken together, it may be that the down-regulation of chromate reductase in resistant lines both limits Mtz activation, and prolongs oxylipin signaling, which could, in turn, promote growth in resistant lines. To test this hypothesis, oxylipins could be added to drug-susceptible trophozoites prior to Mtz exposure, or resistant lines treated with lipoxygenase inhibitors.

A KefF-like quinone reductase encoded by GL_17151, was also down-regulated in all resistant lines investigated here, and was dynamically suppressed in a previous study after exposure of drug-sensitive trophozoites to Mtz (Ansell et al., 2016). These results strongly support a role for GL_17151 in Mtz activation. Putative structural homologs of NADP-binding KefC subunits were also found in our study. In *E. coli* the Kef system couples cytosolic oxidation with pH regulation, which can minimize oxidative damage to biomolecules (Healy et al., 2014). GL_17151 is a paralog of DT-diaphorase, an enzyme that converts oxygen to hydrogen peroxide (Sánchez et al., 2001; Li and Wang, 2006), which is up-regulated in lines 713-r and WB-r. Interestingly, an orthologous enzyme in *T. vaginalis* with H_2_O_2_-forming function, termed flavin reductase-1, is down-regulated in Mtz-resistant lines. Loss of this enzyme in *T. vaginalis* is proposed to confer Mtz resistance by promoting futile redox cycling (Leitsch et al., 2014). The most parsimonious explanation for the contrasting regulation of these orthologs *G. duodenalis* and *T. vaginalis* is that DT-diaphorase can detoxify Mtz, whereas the flavin reductase-1 cannot. Indeed, subtle biochemical differences in paralogous enzymes that markedly affect substrate specificity and reaction stoichiometry are well supported; in the ferredoxin nitroreductases of *G. duodenalis* (Müller et al., 2013, 2015), the canonical nitroreductases in *H. pylori* (Olekhnovich et al., 2009; Martinez-Julvez et al., 2012), and the expanded flavin reductase family in *T. vaginalis* (Leitsch et al., 2014). Effective management of functionally divergent paralogs likely requires concerted transcriptional regulation. Further to this, we found an inverse abundance of sense and antisense transcripts arising from the paralogous chromosomal neighbours GL_17150 and GL_17151, consistent with antisense interference. The role of antisense transcripts could be further probed via episomal overexpression of the pseudogene antisense to GL_17150 (Figure 5B), which is replicated antisense to DT-diaphorase. Transcriptional down-regulation of genes orthologous to GL_9719 and GL_17151 in Mtz-resistant *T. vaginalis*, and the established link between Mtz-resistant *H. pylori* and mutations in nitroreductase-coding genes (Sisson et al., 2002; Olekhnovich et al., 2009; Martinez-Julvez et al., 2012), demonstrate the centrality of FMN-dependent enzymes to Mtz metabolism and resistance across pathogenic species. Detailed investigation of the localization, interacting partners, substrate specificity, and reaction kinetics of these enzymes is now clearly warranted.

### Cysteine-rich membrane proteins

Expression of certain VSPs, the major surface antigens in *G. duodenalis*, is associated with the development of drug resistance (Chen et al., 1995; Müller et al., 2007a, 2008). These cysteine-rich molecules are proposed to protect *G. duodenalis* from environmental stresses such as stomach peptidases, and are constitutively turned over, possibly to evade the host immune system (Nash, 2002). The prominent transcription of 10 VSPs in all resistant lines, suggests positive selection from a more varied VSP population in susceptible trophozoites (Supplementary Figure 5). Whereas these dominant genes seem likely to confer a selective advantage in the presence of Mtz, in our investigations their functional significance has remained elusive. Specifically, we found little difference in the cysteine content and motif number between highly-transcribed VSPs in resistant and susceptible lines, and cysteine substitutions were not highly prevalent. It is possible that resistance-associated VSPs play a role that predominates antioxidant activity, for example, in maximizing the stability and integrity of degraded plasma membranes (Touz et al., 2005). In contrast, the high proportion of CxxC motifs in the consistently up-regulated HCMP GL_11309, supports an important function in protecting membranes from oxidative damage in the presence of Mtz. Functional considerations aside, this molecule and the highly transcribed VSPs in resistant lines, could yet be explored in clinical isolates as potential biomarkers of resistance.

### Isotype-specific resistance mechanisms

The transcriptional changes discussed thus far are common to all three Mtz-resistant lines, and are therefore most likely to account for increased Mtz tolerance. However, it is clear in this study and others, that cellular phenotypes such as growth rate and infectivity diverge between resistant lines (Tejman-Yarden et al., 2011). Those that remain infectious may be more clinically relevant, and thus concerning, than non-infectious resistant lines. The divergent transcriptomes of 106-r and 713-r belie similar growth rates, although only 106-r can establish experimental infections (Tejman-Yarden et al., 2011). Alternatively, despite the transcriptional similarities between 713-r and WB-r, the latter line grows more slowly. Investigating the transcriptional features that are unique to each line can shed some light on the molecular bases of these different cellular phenotypes, and on transcriptional changes that may augment drug resistance.

106-r remains infective, grows relatively well and exhibits the fewest DTGs, which together suggest a highly efficient resistance phenotype. The non-sense mutation in 29% of transcripts encoding nitroreductase-1, could functionally ablate a substantial proportion of the nitroreductase-1 enzyme pool (assuming for simplicity equimolar transcript and protein concentrations), and may thus contribute to the increased Mtz tolerance of 106-r. This finding constitutes the first report of a functional mutation in a known Mtz-activating gene in *G. duodenalis*, which, combined with transcriptional suppression of the same enzyme, should substantially decrease nitroreductase-1 function in 106-r. In addition, 106-r is the only line in which the NAD scavenging pathway and the putative Mtz-detoxification enzyme, nitroreductase-2, are up-regulated. If these changes preference the flow of glycolytic electrons to nitroreductase-2, (either indirectly via NADH, or directly via the ferredoxin electron transport chain as proposed above), then 106-r may essentially use Mtz as a terminal electron acceptor in glycolysis. Such an adaptation would allow sustained PFOR activity and possibly ATP generation via acetyl-CoA, whilst negating the detrimental effects of the activated drug. Continued ATP generation might in turn decrease reliance on other sources of ATP, such as the arginine dihydrolase pathway, which is transcriptionally suppressed.

Increased NAD scavenging in 106-r might also support heightened activity of NAD-dependent Sir2 deacetylases, which are redox-responsive histone modification enzymes (Fulco et al., 2003). By contrast, transcriptional suppression of a Sir2-coding gene (GL_16569) was found in WB-r, and is previously reported in WB-C4 (Müller et al., 2008), a line whose transcriptional profile correlates negatively with 106-r but positively with 713-r and WB-r. It would be interesting to test whether chemical or genetic perturbation of Sir2 enzymes could recapitulate aspects of these divergent molecular resistance phenotypes. Further, 106-r is the only line in which a set of V-type ATPases and a MATE-like transporter are up-regulated. Like other protists, *G. duodenalis* may utilize V-type ATPases at the plasma membrane to regulate cytosolic pH (Biagini et al., 2001a). Increased transcription of dynein and kinesin-related proteins in 106-r is consistent with the recycling of such ATPases via microtubule-dependent transport of ATPase-rich vesicles, evidenced in higher eukaryotes (Breton and Brown, 2013). Active proton efflux, coupled to a sodium:proton exchanger (up-regulated in 106-r and WB-r; Biagini et al., 2001a), may decrease the cytosolic sodium concentration sufficiently to drive Mtz efflux via the MATE transporter, which is a sodium-coupled multidrug efflux pump (Du et al., 2015). Indeed, although MATE transporters are generally associated with extruding cationic compounds, the possibility of Mtz efflux through these channels is supported by experiments in *H. pylori* demonstrating increased Mtz sensitivity MATE-knockout lines (van Amsterdam et al., 2005; Figure 4).

The transcriptome of 713-r is similar to WB-r, despite a marked difference in growth rate. This may derive from active and passive resistance mechanisms that appear unique to 713-r. In addition to lower transcription of nitroreductase-1, a physiological role for NTR-1 in Mtz activation is supported by lower transcription of the encoding gene in this line. Similarly, marked transcriptional suppression of the putative nitroreductase GL_8377, and GL_17150, provides further evidence that several hitherto understudied FMN-binding enzymes may contribute to Mtz activation. Down-regulation of nitroreductase-2 in 713-r may, however, preclude the energetic efficiencies postulated to occur in 106-r. Induction of iron hydrogenase might compensate for lower nitroreductase-2 abundance, and sustain some PFOR activity by directing the flow of electrons to form hydrogen (Lloyd et al., 2002; Figure 3). Further, the marked transcriptional induction of flavohaemoglobin, might represent an alternative Mtz detoxification enzyme. Flavohaemoglobin homologs in bacteria are shown to bind to imidazole drugs (Helmick et al., 2005); and a homolog of this enzyme in the intestinal parasitic protist *Blastocystis hominis*, is postulated to contribute to Mtz resistance (Wu et al., 2014)—findings that warrant further testing of the ability of the *G. duodenalis* flavohaemoglobin to detoxify Mtz.

Despite evidence supporting passive Mtz evasion and active detoxification mechanisms in 713-r, other transcriptional features suggest that this line is under greater oxidative stress than 106-r. Up-regulation of components of the thioredoxin system including TrxR, protein disulfide isomerases-2 and -4 (previously reported in WB-C4; Müller et al., 2008), and peroxiredoxin-1ai, indicate reliance on an antioxidant system that collaterally activates Mtz. It is conceivable that Mtz-induced damage necessitates greater thioredoxin activity, creating a positive feedback loop that constitutes an indirect and metabolically burdensome Mtz detoxification system. Although we did not observe increased transcription of NADPH-forming enzymes that might assist thioredoxin reductase activity in 713-r, the functional significance of the T339A substitution predicted in glucose-6-phosphate dehydrogenase, which catalyzes the first step in the NADPH-forming pentose-phosphate pathway, requires further investigation. Lastly in 713-r, up-regulation of protein chaperones suggests that the thioredoxin system alone is insufficient to manage Mtz-induced damage. Induction of peptidyl-prolyl isomerases, HSPs-90 and TCP-1 chaperonins, may help to maintain the structure and function of damaged proteins, although suppression of proteasomal subunits does not support concomitant recycling of highly degraded proteins. Increased activity of ATP-dependent chaperones may be serviced by induction of glycolytic genes in 713-r, especially the ATP-forming phosphoglycerate kinase. Induction of orthologous peroxidredoxins and ATP-dependent HSPs70 and 90 in Mtz-resistant *G. duodenalis* and *T. vaginalis*, may constitute universal active resistance mechanisms that complement passive down-regulation of FMN-dependent enzymes.

The transcriptional changes unique to, and uniquely lacking in WB-r may account for its slower growth. Lower transcription of iron-binding enzymes including ferredoxin-3, both PFOR paralogs, putative lipoxygenases and a Fe-S cluster scaffold protein, is intriguing given that a key oxygen detoxification enzyme, NADH oxidase (Brown et al., 1996), is down-regulated solely in WB-r. We previously reported transcriptomic results suggesting heightened oxidative stress in WB-s trophozoites at log and stationary phases of *in vitro* culture, when PFOR transcription is comparatively low (Ansell et al., 2015a). As the function of Fe-S-binding enzymes such as PFORs is sensitive to dissolved oxygen (Dan et al., 2000), together these findings suggest that WB-r may endure increased intracellular oxygen, consistent with reliance on futile redox cycling. In this context, concerted induction of genes encoding multiple tRNA biosynthesis enzymes, ribosomal proteins and translation initiation factors, may reflect heightened demand for protein synthesis in WB-r, possibly to replace oxidized proteins. Further, as outlined above, lipoxygenases initiate oxylipin signaling pathways in eukaryotes, and oxylipins, such as prostaglandins are implicated in sustaining the growth and development of other protists (Noverr et al., 2003). If the heightened oxidative stress postulated within WB-r is not compatible with sustained expression of iron-dependent lipoxygenases, then a scarcity of oxylipins may compound the observed growth defect. This phenotype may also relate to insufficient induction of important genes in WB-r, relative to the other resistant lines. For example, inorganic sulfate is liberated from sulfate esters by sulfatases, which require sulfatase maturating enzymes to become catalytically active (Bojarová and Williams, 2008). Although a sulfatase-like enzyme is up-regulated in all lines, the Fe-S-dependent maturating enzyme is only up-regulated in 106-r and 713-r. If sulfur metabolism is less efficient in WB-r as a result, then non-protein antioxidant activity could also be impaired (Brown et al., 1998). More broadly, transcripts that were exclusively detected in 106 and 713 isotypes, such as a highly transcribed putative tRNA methyltransferase which may modulate translation efficiency; or those which were only detected the WB isotype, may also contribute to the phenotypic differences between resistant lines. Future work to detail the genetic differences between these isotypes, and between the resistant and susceptible lines therein, will be necessary to differentiate those transcriptional differences based in genetic architecture, from those based purely in dynamic transcriptional regulation. Although the infectivity of WB-r has not been tested, we expect that this line would lack infectivity due to both the similarity between its transcriptional phenotype and that of the non-infective 713-r, and its retarded growth.

## Concluding remarks

The Mtz-resistant lines 106-r, 713-r, and WB-r are among the best-characterized *G. duodenalis* lines, as subjects of biochemical and genetic research over the past 30 years. The present study combined deep sequencing with protein structure prediction to profile the transcriptional foundations of the biochemical and cellular phenotypes of these seminal lines. Previously, we contended that the handful of enzymes known to metabolize Mtz, need to be considered in the context of the vast metabolic networks which support their function. We now identify networks of carbon flux, thiol cycling, and NAD synthesis, among others, which are differentially regulated in Mtz-resistant *G. duodenalis*, and propose important roles for under-studied enzymes. Passive resistance mechanisms that are common among the three resistant lines include transcriptional down-regulation of nitroreductase-1, and of enzymes that are likely to support PFOR activity. Functionally analogous changes in different resistant lines that conserve free acetyl-coA and thus pyruvate, might suppress PFOR activity and bolster pyruvate-dependent antioxidant activity. Down-regulation of hitherto under-studied FMN-binding enzymes, including putative quinone and chromate reductases as well as canonical nitroreductases, may constitute novel passive resistance mechanisms, which are emulated in *T. vaginalis*. The transcription of these enzymes appears tightly regulated, possibly via antisense interference, which may relate to subtle biochemical differences between paralogues that exert opposing effects on Mtz toxicity. Convergence among highly transcribed VSPs in resistant lines is consistent with a selective advantage in the presence of Mtz, which transcends global cysteine content or motif number. Cysteine sparging capacity may, however, be under selection in high cysteine membrane proteins.

Molecular changes are of particular interest in 106-r, as this line remains infectious. Up-regulation of nitroreductase-2, together with a nonsense mutation in nitroreductase-1, and induction of a putative MATE-like drug efflux system, together suggest a highly metabolically efficient resistance phenotype. Incidentally, the strength of correlation between transcriptional changes in trophozoites in lines 106-r, and in WB-s after exposure to sub-lethal Mtz, further highlights the potential clinical relevance of laboratory models for investigating drug response and resistance. It appears that 713-r and WB-r are more reliant on active management of Mtz- and oxygen-induced damage, respectively. The former is consistent with collateral activation of Mtz via the thioredoxin system; the latter with Mtz inactivation through passive futile cycling. Increased glycolysis may be necessary to sustain these energetically expensive strategies, and failure to up-regulate genes encoding glycolytic or sulfur cycling enzymes, might account for the sluggish growth of WB-r. Future work to correlate transcript and protein abundances in these resistant lines should assess the contribution of post-transcriptional regulation to Mtz resistance, and reveal the functional penetrance of the amino acid mutations predicted here. Pursuing these avenues would do much to establish *G. duodenalis* as the defining model for understanding, and ultimately for preventing, Mtz resistance in microaerophilic eukaryotic pathogens.

## Author contributions

BA, LB, and AJ designed experiments. BA and LB generated experimental samples. BA, SS, and AJ analyzed data. BA, SE, MM, RG, SS, and AJ wrote the manuscript.

## Funding

This work, including the efforts of AJ and MM, was funded by Australian Research Council (ARC) (LP120200122). BA, LB, SE, and AJ are supported by the Victorian State Government Operational Infrastructure Support and Australian Government National Health and Medical Research Council Independent Research Institute Infrastructure Support Scheme. BA is supported by an Australian Postgraduate Award (Australian Government) and the Victorian Life Sciences Computation Initiative (Victoria, Australia). RNA sequencing was partially funded by YourGene Biosciences (Taiwan). The funders had no role in study design, data collection and interpretation, or the decision to submit the work for publication.

### Conflict of interest statement

The authors declare that the research was conducted in the absence of any commercial or financial relationships that could be construed as a potential conflict of interest.

## Supplementary material

Supplementary Table 1 **Sense and antisense read mapping statistics for metronidazole resistant (-R) and susceptible (-S) lines**.

Supplementary Table 2 **TMM-normalized counts per million counts (CPM) values for all replicates**.

Supplementary Table 3 **Coding transcripts detected in resistant and susceptible trophozoites from one or two isotypes**.

Supplementary Table 4 **Transcriptional abundance, fold-change, and functional annotations for genes detected in all resistant and susceptible *Giardia* trophozoite lines**.

Supplementary Table 5 **Transcriptional abundance, fold-change, and functional annotations for 156 consistently up- or down-regulated genes**.

Supplementary Table 6 **Transcriptional abundance and functional annotations for the 30 most strongly up- and down-regulated genes in each resistant line, excluding variant-specific surface proteins (VSPs)**.

Supplementary Table 7 **Synonymous and non-synonymous single nucleotide polymorphisms (SNPs) and predicted peptide modifications in resistant lines, relative to susceptible parent lines of the same genotype**.

Supplementary Table 8 **Comparative transcription and single nucleotide polymorphism (SNP) statistics for transcripts encoding variant-specific surface proteins (VSPs)**. Transcripts among the top decile (20) of highly transcribed VSPs in susceptible and resistant lines are identified. Clade and cysteine content information is adapted from Adam et al. (2010) doi: 10.1186/1471-2164-11-424.

Supplementary Table 9 **Curated gene sets specific to *Giardia duodenalis* and used in rotational gene enrichment testing**.

Supplementary Table 10 **KEGG terms enriched among differentially transcribed genes in resistant- relative to susceptible trophozoite lines, with comparative transcriptional statistics for contributing genes**.

Supplementary Table 11 **Gene Ontology (GO) Molecular Function terms enriched among differentially transcribed genes in resistant- relative to susceptible trophozoite lines, with comparative transcriptional statistics for contributing genes**.

Supplementary Table 12 **Gene Ontology (GO) Biological Process terms enriched among differentially transcribed genes in resistant- relative to susceptible trophozoite lines, with comparative transcriptional statistics for contributing genes**.

Supplementary Table 13 **Comparative transcription statistics for non-overlapping, non-deprecated antisense transcripts, with differential transcription statistics for the corresponding sense transcripts**.

Supplementary Table 14 **Comparative transcription statistics for non-overlapping, non-deprecated antisense transcripts that are differentially transcribed in the same direction in all resistant lines**.

Supplementary Table 15 **Orthologous differentially transcribed genes metronidazole-resistant *Giardia duodenalis* and *Trichomonas vaginalis***.

Supplementary Figure 1 **Saturating RNA sequencing depth**. Total and novel transcript detection as a function of read depth for reads mapping in the sense orientation to accepted gene models. Different colors represent biological replicates.

Supplementary Figure 2 **Transcriptome clustering for metronidazole-resistant and -susceptible lines. (A)** Primary principal co-ordinate clustering based on biological coefficient of variance (BCV) for resistant and susceptible lines. Susceptible lines are represented in in soft colors, and shaded. **(B)** Unsupervised clustering of z-scaled transcript abundance values (by row) for biological triplicates (columns). Resistant and susceptible lines cluster separately. **(C)** Mean Pearson correlation coefficients (*r*) for resistant and susceptible transcriptomes with (top), and without 200 variant-specific surface protein (VSP)-coding genes (bottom).

Supplementary Figure 3 **Single nucleotide polymorphisms and predicted amino acid substitutions**. Thirty-nine non-synonymous SNPs were identified in in coding transcripts in 106-r, and 211 and 70 such SNPs were identified in 713-r and WB-r respectively, which may relate to preliminary UV mutagenesis of the latter lines. Non-synonymous SNPs were predicted to bias the proteomes of resistant lines toward alanine, cysteine and glutamine residues, and to decrease the proportion of asparagine. **(A)** Net changes in amino acid prevalence in metronidazole-resistant lines relative to susceptible parents (at left; WB-r: red; 106-r: green; 713-r: blue), and each amino acid as a proportion of all substituted amino acids in each resistant line (right). **(B)** Mapped synonymous and non-synonymous SNPs as a proportion of all nucleotides mapped at the relevant locus, when a lower threshold of 20% prevalence for the alternative allele is applied. Partial prevalence is evident for most SNPs.

Supplementary Figure 4 **Highly transcribed variant-specific surface protein-coding genes (*vsps*) in metronidazole-susceptible and -resistant *Giardia duodenalis*. (A)** Overlap in the 20 most highly transcribed *vsp*s in susceptible (left) and resistant (right) lines. Ten *vsps* that are highly transcribed in all three resistant lines are marked with an asterisk in panel **(B)**. **(B)** Unsupervised clusters formed by 32 *vsp*s that are among the top 20 most highly transcribed *vsp*s in at least one susceptible, or resistant line. Colors represent TPM values, z-scaled by row.

Supplementary Figure 5 **Transcriptional changes in genes encoding high-cysteine membrane proteins in metronidazole-resistant lines**. Log_2_(fold change) values are represented as colors. GL50803_11309 is the only HCMP-coding gene that is significantly up-regulated in all three lines.

Supplementary Figure 6 **Nonsense mutation in transcripts encoding nitroreductase-1 (GL50803_22677) in 106-r**. Screen capture from Integrative Genomics Viewer. Triplicate tracks are shown for 106-r (top) and 106-s (bottom). Aligned reads are displayed in mauve. A single nucleotide polymorphism (C538T; red) is visible in the top tracks.

Supplementary Figure 7 **Predicted protein structure for the putative chromate reductase (GL50803_9719)**. The putative GL50803_9719 structure, at left, is similar to the Old Yellow Enzyme from *Thermus scotoductus* (at centre; RMSD = 1.06 Å; PDB code 3hf3; Opperman et al., 2010) and the chromate reductase from *Bacillis subtilis* (at right; RMSD = 1.32 Å; PDB code 1z48; Kitzing et al., 2005). The FMN cofactor and structural sulfate in the crystal structures, are displayed in red.

Supplementary Figure 8 **Change in transcription of orthologous genes in Mtz-resistant *Giardia duodenalis* and *Trichomonas vaginalis***. Two-sample comparisons of transcription in Mtz-resistant and –susceptible *G. duodenalis* (circles) and *T. vaginalis* (triangles) revealed differentially transcribed orthologs from eighteen different groups (key, right), with genes of interest annotated. For clarity, the orthologous group OG5_126566, which contains four *G. duodenalis* proteins and 41 *T. vaginalis* ankyrin repeat-like proteins, is omitted from the chart. Full gene descriptions for each group are provided in Supplementary Table 15. CPM, counts per million counts; FC, fold change; HSP, heat shock protein; UDP, uridine diphosphate.
