# Supplementary figures and images for: Transcriptomics Indicates Active and Passive Metronidazole Resistance Mechanisms in Three Seminal Giardia Lines
Source: Front Microbiol. 2017 Mar 17;8:398. doi: 10.3389/fmicb.2017.00398 (PMC5355454; doi:10.3389/fmicb.2017.00398)

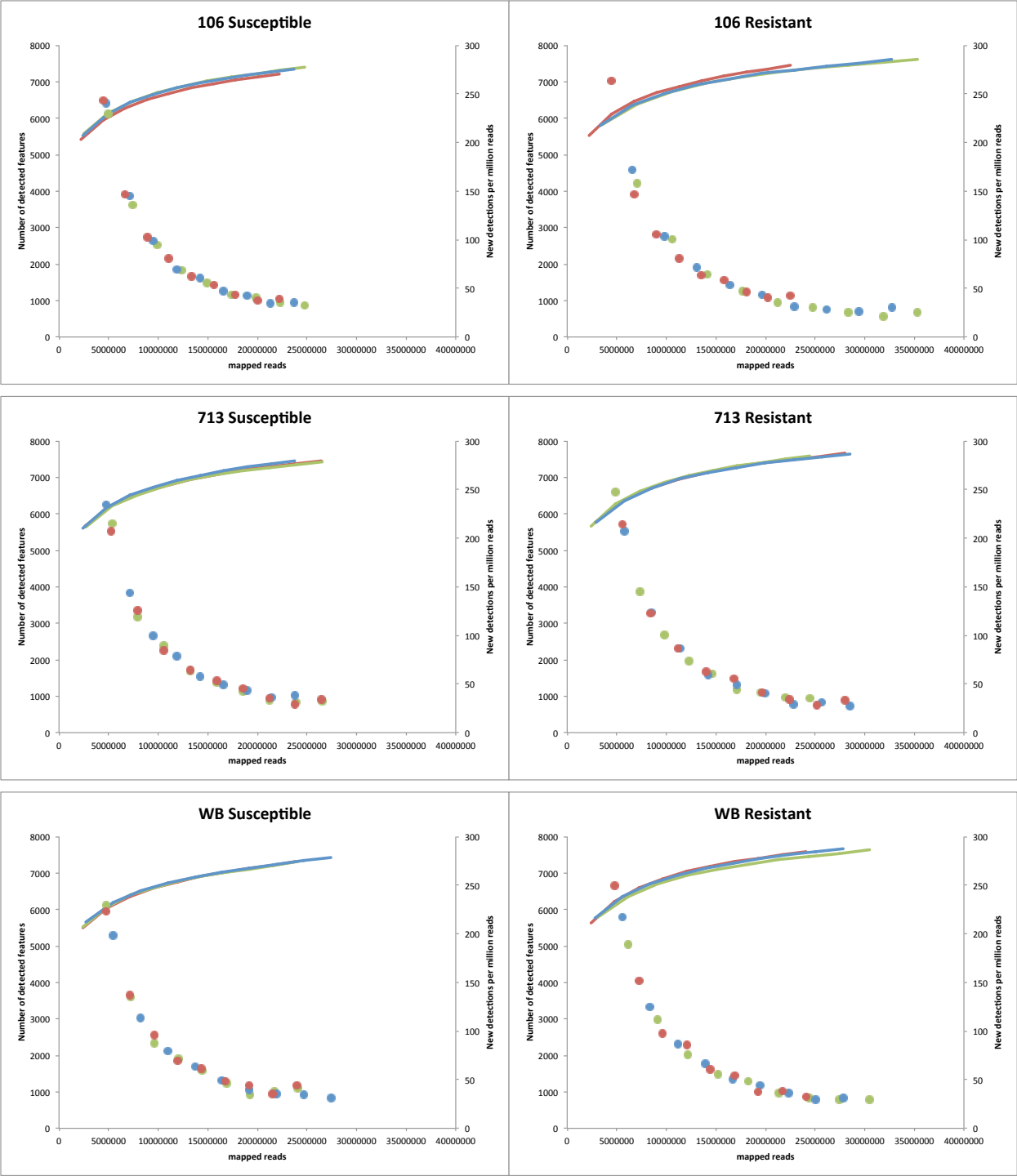

Supplement: Supplementary Figure 1 — Saturating RNA sequencing depth. Total and novel transcript detection as a function of read depth for reads mapping in the sense orientation to accepted gene models. Different colors represent biological replicates. [file Image1.pdf]

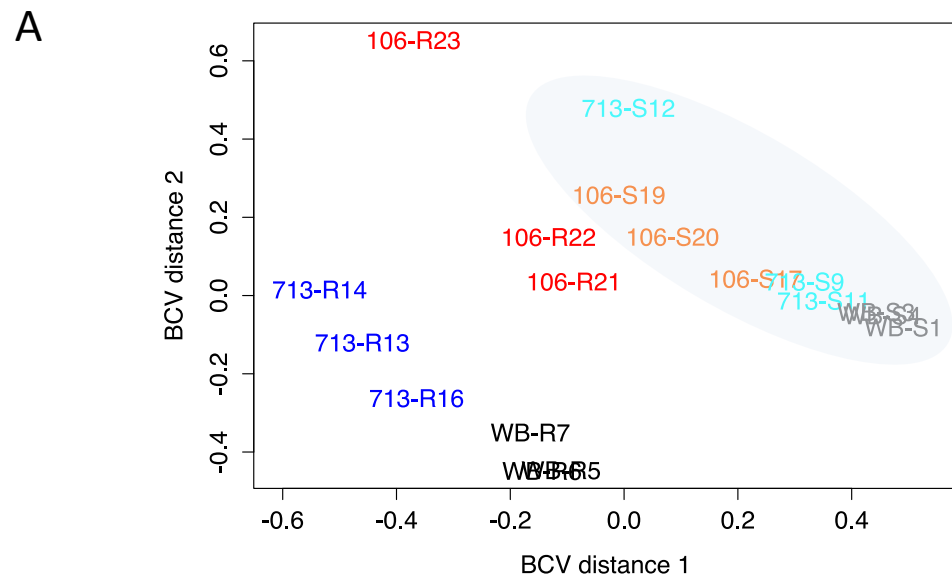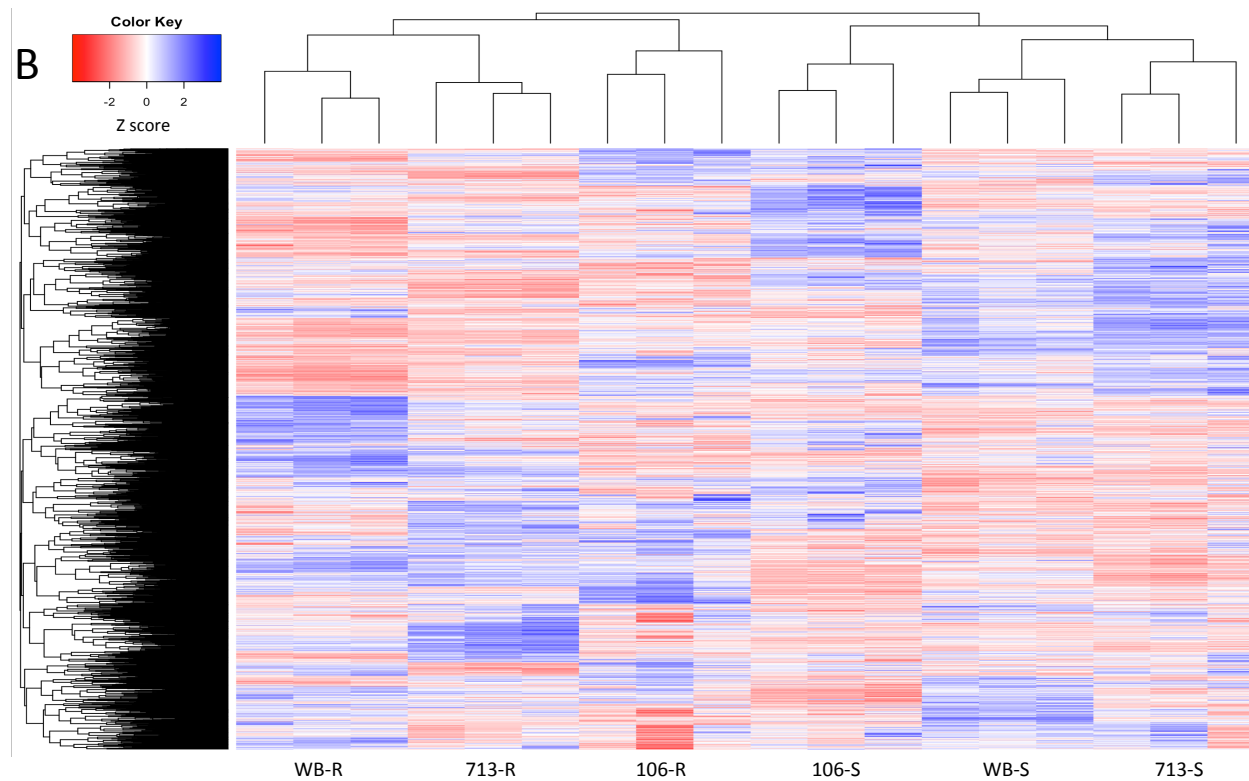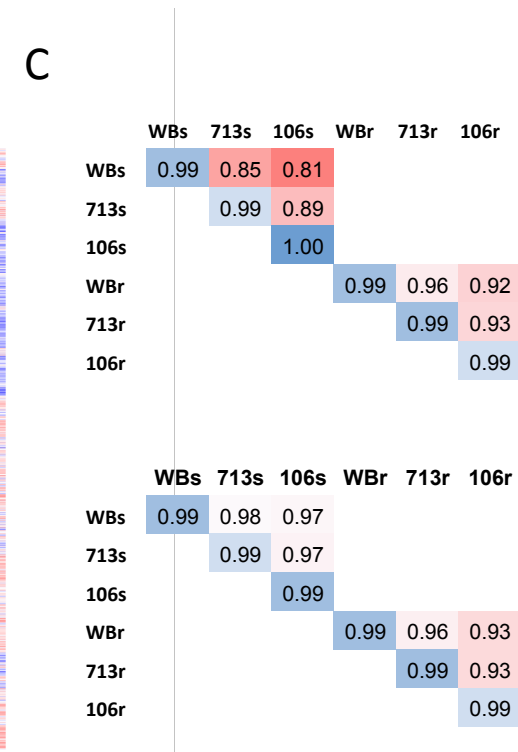

Supplement: Supplementary Figure 2 — Transcriptome clustering for metronidazole-resistant and -susceptible lines. (A) Primary principal co-ordinate clustering based on biological coefficient of variance (BCV) for resistant and susceptible lines. Susceptible lines are represented in in soft colors, and shaded. (B) Unsupervised clustering of z-scaled transcript abundance values (by row) for biological triplicates (columns). Resistant and susceptible lines cluster separately. (C) Mean Pearson correlation coefficients (r) for resistant and susceptible transcriptomes with (top), and without 200 variant-specific surface protein (VSP)-coding genes (bottom). [file Image2.pdf]

A

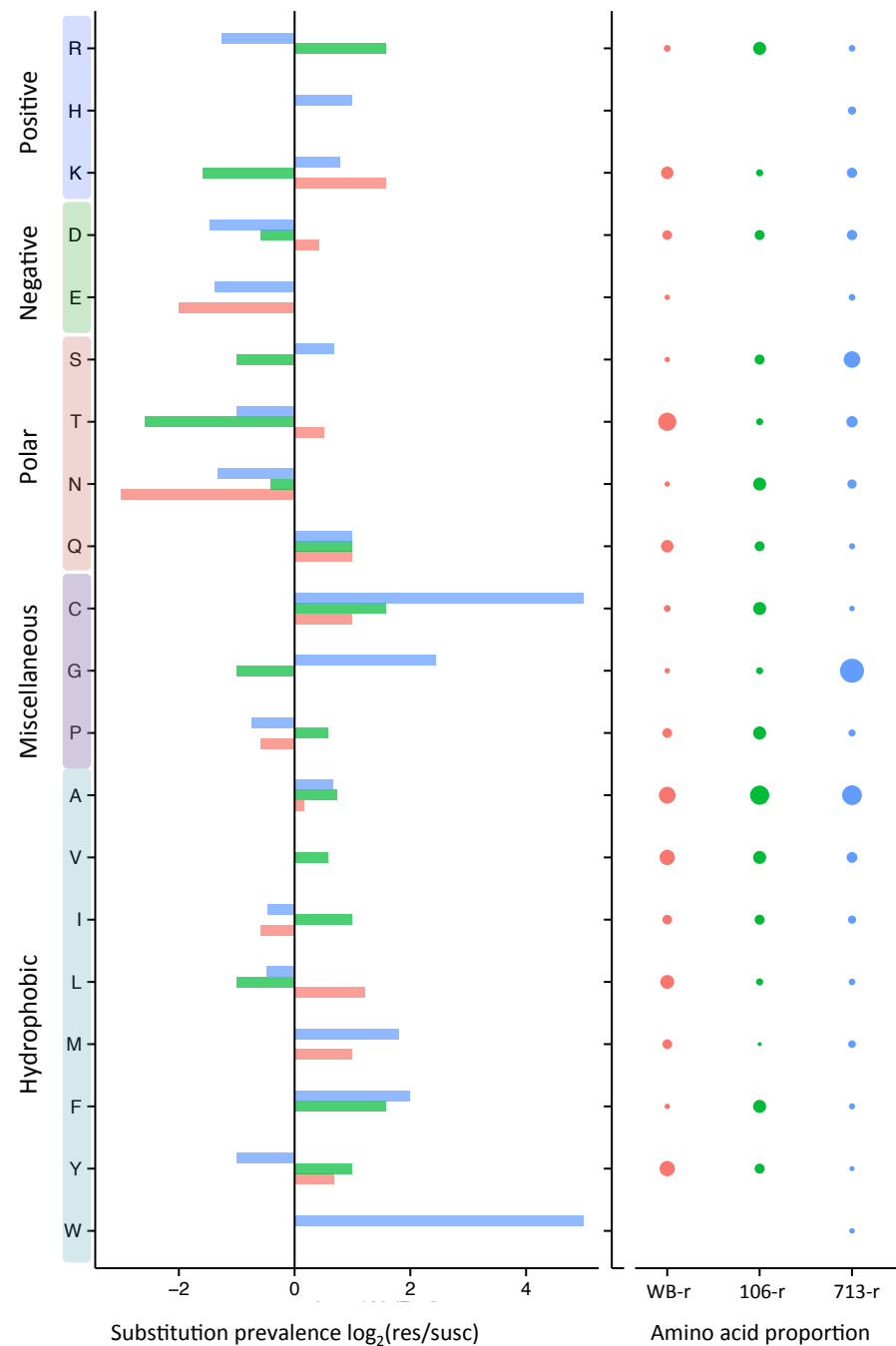

B

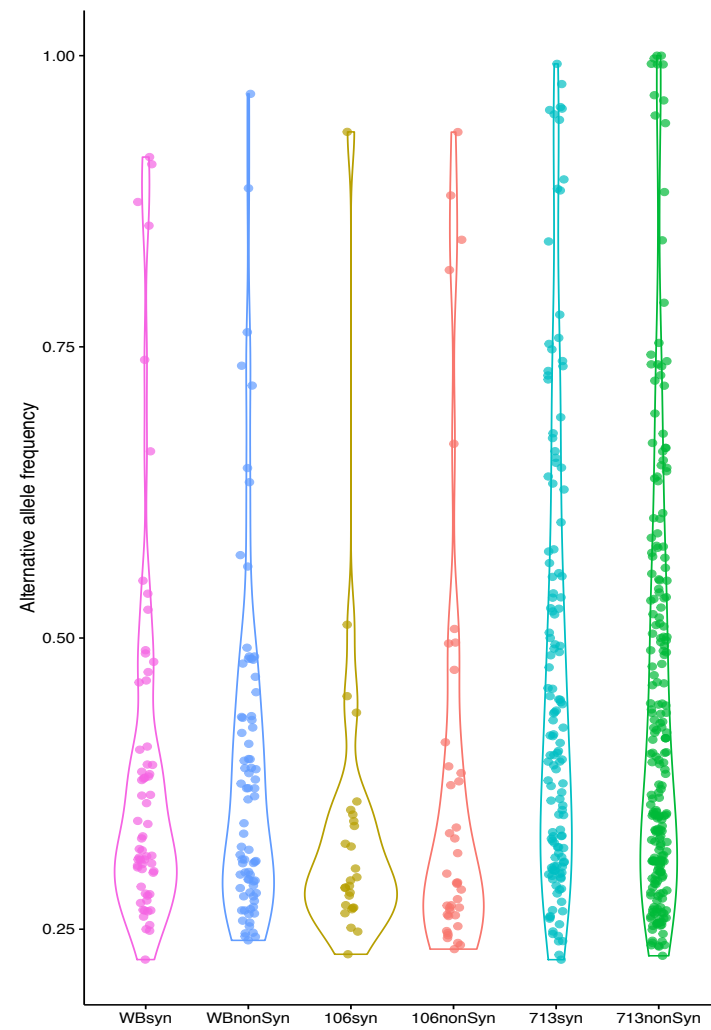

Supplement: Supplementary Figure 3 — Single nucleotide polymorphisms and predicted amino acid substitutions. Thirty-nine non-synonymous SNPs were identified in in coding transcripts in 106-r, and 211 and 70 such SNPs were identified in 713-r and WB-r respectively, which may relate to preliminary UV mutagenesis of the latter lines. Non-synonymous SNPs were predicted to bias the proteomes of resistant lines toward alanine, cysteine and glutamine residues, and to decrease the proportion of asparagine. (A) Net changes in amino acid prevalence in metronidazole-resistant lines relative to susceptible parents (at left; WB-r: red; 106-r: green; 713-r: blue), and each amino acid as a proportion of all substituted amino acids in each resistant line (right). (B) Mapped synonymous and non-synonymous SNPs as a proportion of all nucleotides mapped at the relevant locus, when a lower threshold of 20% prevalence for the alternative allele is applied. Partial prevalence is evident for most SNPs. [file Image3.pdf]

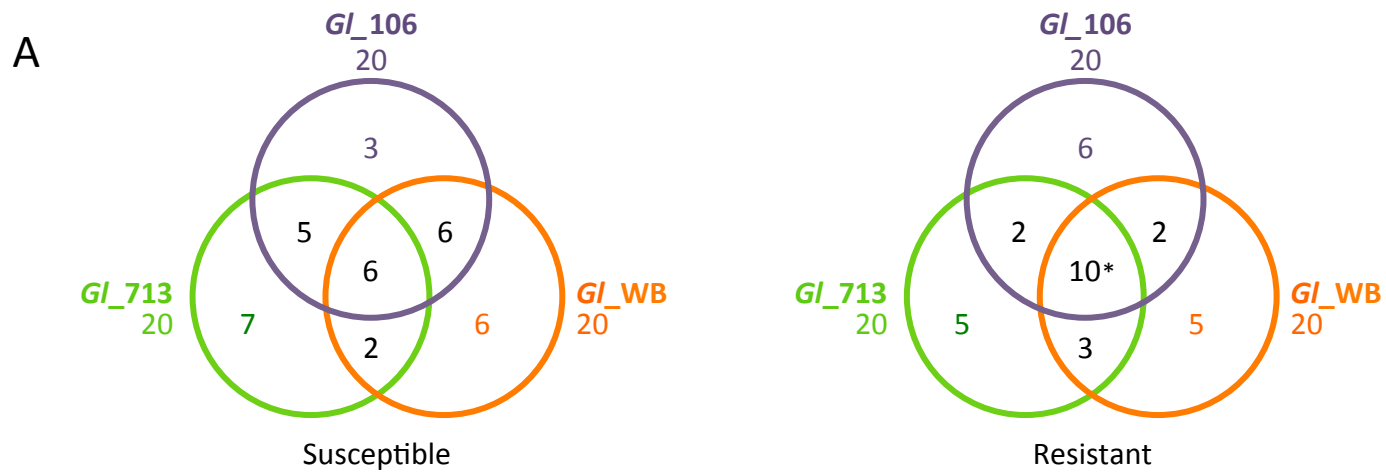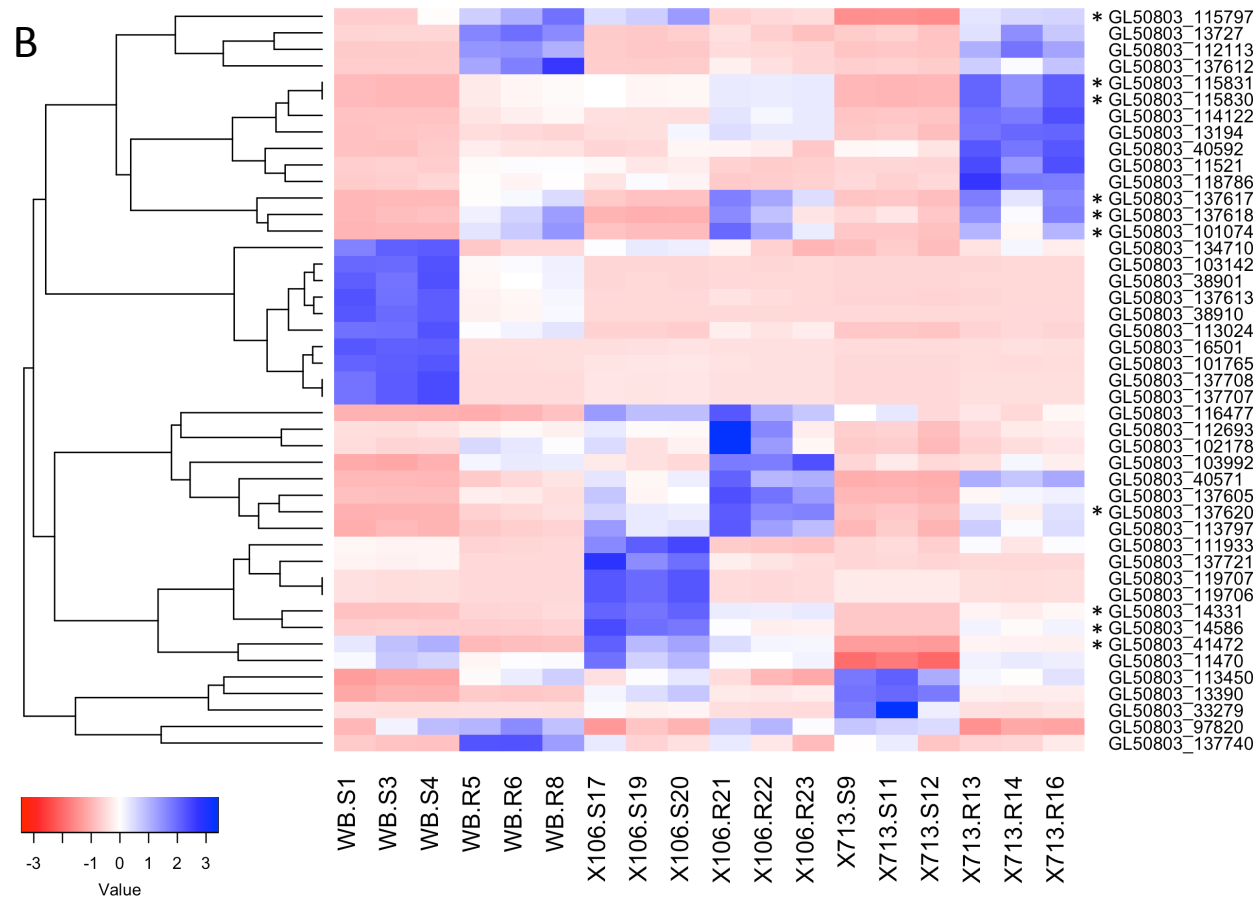

Supplement: Supplementary Figure 4 — Highly transcribed variant-specific surface protein-coding genes (vsps) in metronidazole-susceptible and -resistant Giardia duodenalis. (A) Overlap in the 20 most highly transcribed vsps in susceptible (left) and resistant (right) lines. Ten vsps that are highly transcribed in all three resistant lines are marked with an asterisk in panel (B). (B) Unsupervised clusters formed by 32 vsps that are among the top 20 most highly transcribed vsps in at least one susceptible, or resistant line. Colors represent TPM values, z-scaled by row. [file Image4.pdf]

Color Key

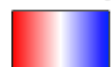

-2 2

Value

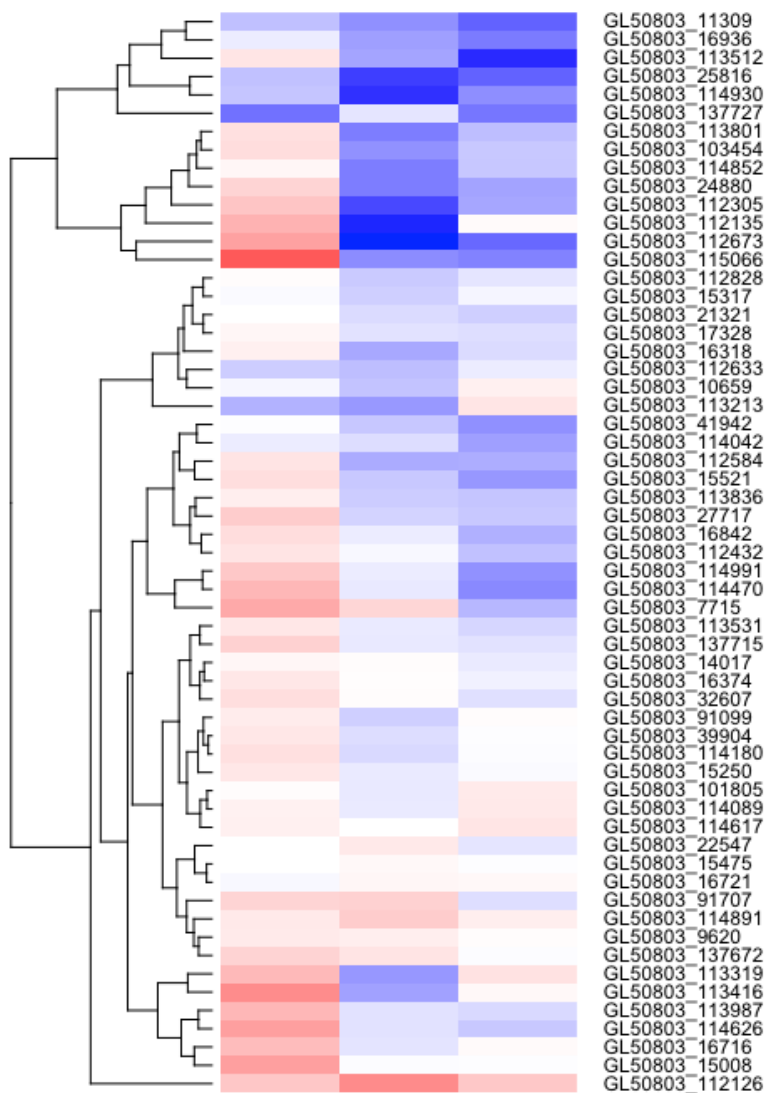

106-r 713-r WB-r

Supplement: Supplementary Figure 5 — Transcriptional changes in genes encoding high-cysteine membrane proteins in metronidazole-resistant lines. Log2(fold change) values are represented as colors. GL50803_11309 is the only HCMP-coding gene that is significantly up-regulated in all three lines. [file Image5.pdf]

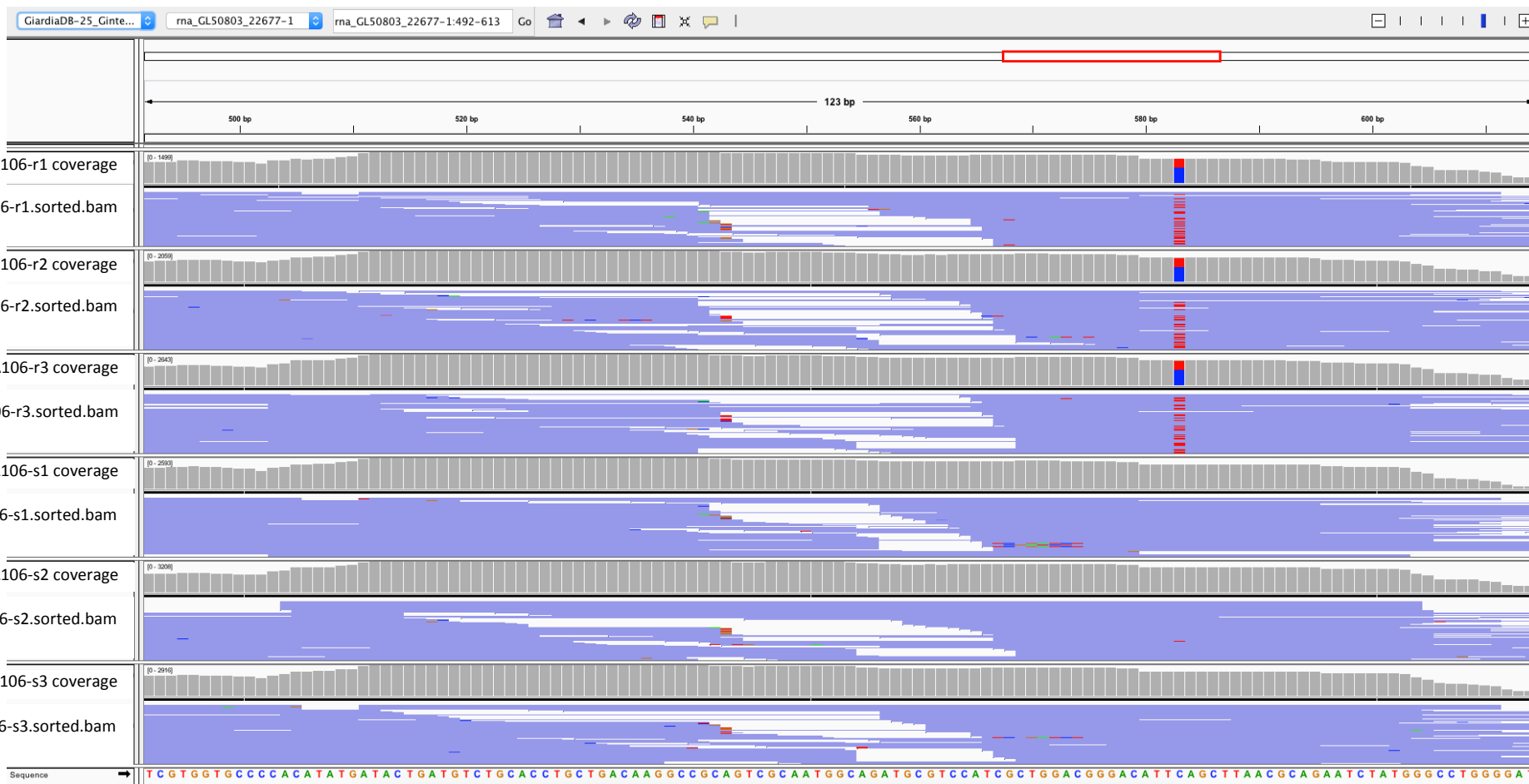

Supplement: Supplementary Figure 6 — Nonsense mutation in transcripts encoding nitroreductase-1 (GL50803_22677) in 106-r. Screen capture from Integrative Genomics Viewer. Triplicate tracks are shown for 106-r (top) and 106-s (bottom). Aligned reads are displayed in mauve. A single nucleotide polymorphism (C538T; red) is visible in the top tracks. [file Image6.pdf]

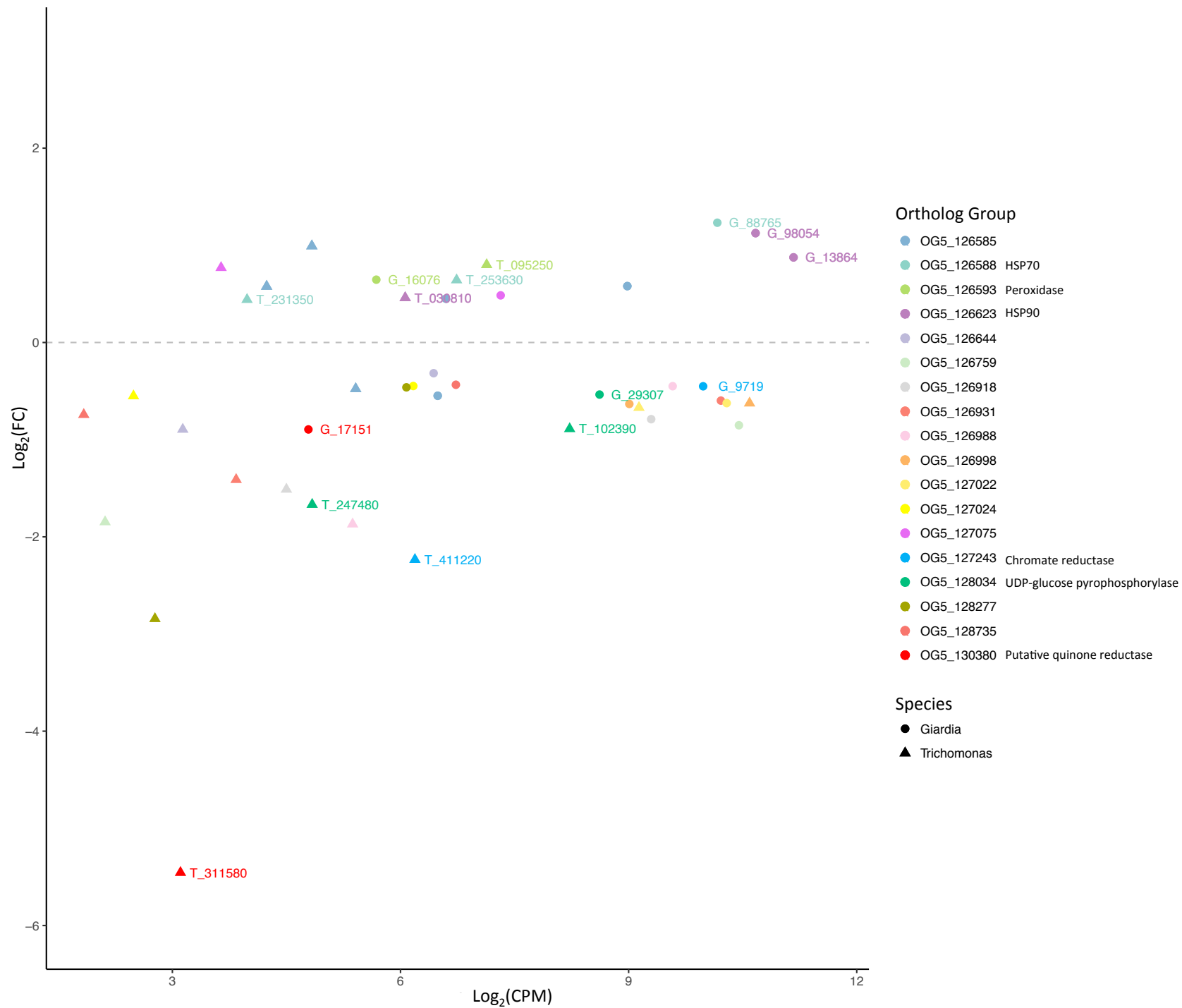

Supplement: Supplementary Figure 8 — Change in transcription of orthologous genes in Mtz-resistant Giardia duodenalis and Trichomonas vaginalis. Two-sample comparisons of transcription in Mtz-resistant and –susceptible G. duodenalis (circles) and T. vaginalis (triangles) revealed differentially transcribed orthologs from eighteen different groups (key, right), with genes of interest annotated. For clarity, the orthologous group OG5_126566, which contains four G. duodenalis proteins and 41 T. vaginalis ankyrin repeat-like proteins, is omitted from the chart. Full gene descriptions for each group are provided in Supplementary Table 15. CPM, counts per million counts; FC, fold change; HSP, heat shock protein; UDP, uridine diphosphate. [file Image8.pdf]
