# Supplementary material for: Transcriptomics Indicates Active and Passive Metronidazole Resistance Mechanisms in Three Seminal Giardia Lines
Source: Front Microbiol. 2017 Mar 17;8:398. doi: 10.3389/fmicb.2017.00398 (PMC5355454; doi:10.3389/fmicb.2017.00398)

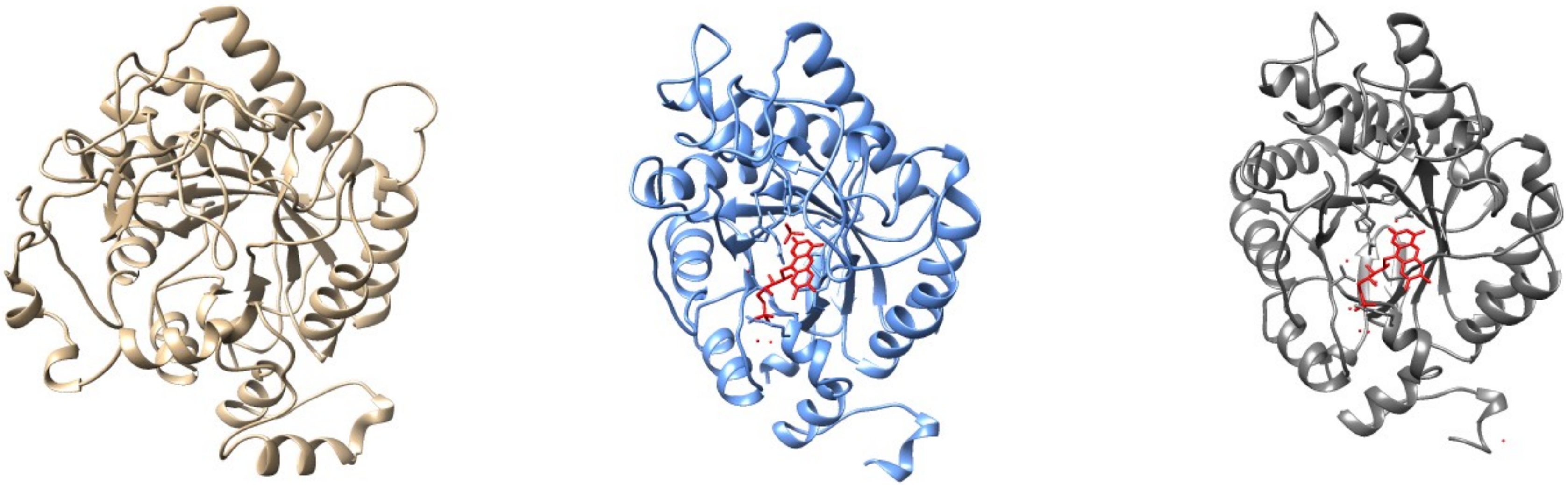

Supplement: Supplementary Figure 7 — Predicted protein structure for the putative chromate reductase (GL50803_9719). The putative GL50803_9719 structure, at left, is similar to the Old Yellow Enzyme from Thermus scotoductus (at centre; RMSD = 1.06 Å; PDB code 3hf3; Opperman et al., 2010) and the chromate reductase from Bacillis subtilis (at right; RMSD = 1.32 Å; PDB code 1z48; Kitzing et al., 2005). The FMN cofactor and structural sulfate in the crystal structures, are displayed in red. [file Image7.pdf]
